# Supplementary material for: Transcriptome Profile Analysis of Intestinal Upper Villus Epithelial Cells and Crypt Epithelial Cells of Suckling Piglets
Source: Animals (Basel). 2022 Sep 7;12(18):2324. doi: 10.3390/ani12182324 (PMC9494997; doi:10.3390/ani12182324)
Supplement: Supplementary file 1 [file animals-12-02324-s001.zip › Table S1.The up-regulation DEGs of F3 compared with F1.pdf]

Table S1. The up-regulation DEGs of the crypt cells (F3) compared with the villus upper cells (F1)

Gene ID	F1 FPKM	F3 FPKM	log2(F3/F1)	Qvalue(F1-vs-F3)	Pvalue(F1-vs-F3)	Other Gene ID
100037933	0.038	1.188	4.686499063	1.46E-18	5.75E-19	IL17F
100037941	2.256	9.822	2.008349991	4.79E-210	2.52E-211	PDE4B
100037943	221.46	484.198	1.06604968	0	0	RETNLB
100038004	2.02	7.866	1.70141258	2.84E-111	2.66E-112	EGR2
100048932	44.844	168.352	1.878623303	0	0	BTG2
100126238	8.964	17.646	1.002182234	2.14E-117	1.90E-118	BCAS1
100144467	2.476	5.31	1.083158033	2.91E-15	1.33E-15	ASCL2
100144477	0.04	0.174	2.200671794	5.67E-05	5.41E-05	CD163L1
100144486	147.534	504.432	1.769091117	0	0	FOS
100144503	42.984	134.418	1.773992339	0	0	RHOB
100144619	59.396	127.194	1.083239249	0	0	ITLN2
100151888	1.35	2.964	1.15831308	1.76E-60	2.72E-61	ZBTB10
100151939	0.038	0.37	2.301413452	5.10E-05	4.84E-05	LYPD1
100151994	1.772	3.422	1.03050231	2.91E-29	8.17E-30	LGR5
100152141	0.898	3.024	1.76685656	2.68E-38	6.01E-39	CTGF
100152200	14.77	34.962	1.173634072	0	0	C7H6orf222
100152253	2.27	5.164	1.175398082	9.82E-37	2.28E-37	STX11
100152349	0.502	1.212	1.193202549	1.80E-12	9.40E-13	AMIGO2
100152492	0.034	0.106	1.627605374	0.000810702	0.000899544	A2ML1
100152504	0.222	0.97	2.057142443	0.000166599	0.000167671	IFN-ALPHA-15
100152997	4.224	13.904	1.722983422	2.73E-78	3.50E-79	GADD45G
100153074	0.544	1.516	1.423464657	1.31E-09	8.22E-10	NCR2
100153176	4.178	8.562	1.05780268	6.45E-25	2.05E-25	SFRP5
100153265	2.362	6.062	1.338098756	3.66E-51	6.52E-52	SH2D2A
100153272	0.19	0.534	1.468907628	0.000221151	0.000226515	RIPPLY3
100153365	3.624	12.218	1.763982232	5.43E-127	4.43E-128	CD83
100153791	0.528	1.294	1.242315218	3.11E-09	2.01E-09	CYR61
100154053	4.868	11.584	1.24979105	3.00E-147	2.11E-148	LOC100154053
100154253	0.11	0.274	1.524511881	2.61E-07	1.97E-07	BICC1
100154395	0.388	0.89	1.201072235	9.95E-08	7.25E-08	NOV
100155114	2.278	4.896	1.137990749	4.12E-33	1.04E-33	TAGAP
100155581	3.976	8.5	1.102943383	6.78E-24	2.21E-24	LTB
100155738	0.018	0.088	2.212567874	0.000110659	0.000109239	LOC100155738

Gene ID	F1 FPKM	F3 FPKM	log2(F3/F1)	Qvalue(F1-vs-F3)	Pvalue(F1-vs-F3)	Other Gene ID
100155836	1.11	2.32	1.087036992	2.24E-18	8.94E-19	SERPINB5
100156484	11.706	32.556	1.45599154	3.15E-291	1.22E-292	MAFF
100156553	0.144	0.392	1.427306723	1.29E-07	9.51E-08	LOC100156553
100156741	0.854	2.48	1.563043917	1.35E-05	1.20E-05	LOC100156741
100156849	0.116	0.318	1.562017032	0.000115316	0.000114121	DISC1
100157298	7.77	17.986	1.208437864	2.88E-122	2.46E-123	SAMSN1
100157564	0.266	0.504	1.004168725	0.000322239	0.000338085	CEBPE
100157952	0.532	1.436	1.471486172	7.60E-16	3.36E-16	HEY1
100174954	3.982	8.174	1.028143303	6.24E-75	8.19E-76	KLF11
100174959	1.004	3.776	1.899608243	2.05E-36	4.80E-37	KLF2
100174961	24.552	51.112	1.05019874	0	0	KLF6
100240743	16.932	58.072	1.676171909	2.31E-217	1.17E-218	DDIT3
100271930	3.936	13.764	1.715961248	1.98E-131	1.57E-132	IL7R
100286800	298.612	801.834	1.41639137	0	0	BPI
100286870	0.162	0.732	2.113032201	1.37E-08	9.33E-09	TNFAIP6
100312980	0.562	1.134	1.089948588	0.000423534	0.000450681	RNASE1
100316849	91.576	258.2	1.480747581	0	0	ZFP36
100337659	1.128	2.776	1.299585531	1.17E-42	2.40E-43	EPHB3
100462720	0.026	0.106	2.244276734	0.000301905	0.000315362	ATG9B
100499568	0.084	0.352	1.968642291	2.24E-05	2.04E-05	HOXA5
100510896	5.932	12.588	1.07470907	3.24E-64	4.76E-65	UBALD1
100511294	0.064	0.164	1.448635232	5.34E-05	5.09E-05	PTPRZ1
100511325	0.704	1.966	1.401096844	3.44E-14	1.65E-14	SLAMF1
100511493	0.016	0.114	2.626001488	0.000454873	0.000486203	GDF1
100511902	5.148	11.842	1.191397174	5.72E-87	6.52E-88	RCAN1
100512029	2.248	4.73	1.072544359	4.55E-34	1.12E-34	SYP
100512063	0.584	1.268	1.106554637	7.03E-05	6.78E-05	POU2AF1
100512231	0.226	0.588	1.502074491	0.000915011	0.001022177	MFAP5
100512416	4.914	10.636	1.120896699	6.46E-56	1.06E-56	FOXA3
100512496	0.616	2.31	1.912582332	1.62E-22	5.52E-23	RASD1
100512960	24.666	55.268	1.186845541	0	0	TRIB1
100514273	6.698	15.904	1.246423982	1.29E-76	1.67E-77	GIF
100514348	6.762	14.524	1.112223065	4.56E-86	5.23E-87	REP15
100514823	0.316	0.654	1.052228922	1.40E-07	1.03E-07	NLRP3

Gene ID	F1 FPKM	F3 FPKM	log2(F3/F1)	Qvalue(F1-vs-F3)	Pvalue(F1-vs-F3)	Other Gene ID
100515419	1.07	3.5	1.673617771	6.96E-20	2.60E-20	CD28
100515679	0.144	0.372	1.318974101	4.03E-05	3.78E-05	CXCR5
100516016	0.13	0.772	2.5280697	2.22E-05	2.02E-05	CCL22
100516472	0.004	0.052	3.743082591	0.000461857	0.000494405	KIAA1755
100516931	0.1	0.536	2.364570968	2.65E-07	2.00E-07	ARL4D
100517416	2.328	4.742	1.014445981	2.90E-36	6.80E-37	DUSP10
100517477	0.414	0.892	1.072390216	1.50E-05	1.34E-05	LOC100517477
100517790	0.17	0.402	1.273968419	0.000205659	0.000209723	CCNO
100517891	1282.366	3208.95	1.312727649	0	0	LOC100517891
100518506	3.442	7.02	1.002179497	9.63E-41	2.05E-41	FAM177B
100518720	0.19	0.366	1.002750146	1.85E-05	1.67E-05	CHSY3
100519286	0.05	0.406	2.779608467	1.12E-07	8.21E-08	SERPINB2
100519542	0.102	0.248	1.219520635	0.000413721	0.00043934	COL26A1
100519728	0.27	0.544	1.014179409	2.34E-06	1.92E-06	KL
100520167	0.108	0.226	1.111684517	3.38E-06	2.82E-06	PAX5
100520241	0.094	0.362	1.890639779	5.67E-05	5.42E-05	LOC100520241
100520336	0.334	0.956	1.499991177	1.18E-13	5.81E-14	NMUR1
100520600	2.754	5.768	1.019315603	3.72E-41	7.87E-42	TP53I11
100520726	47.034	199.478	2.073630317	0	0	EGR1
100520768	0.136	0.842	2.581801684	8.29E-15	3.85E-15	CCDC173
100520915	1.036	3.566	1.632924071	2.55E-88	2.87E-89	COL11A2
100520981	22.736	55.004	1.094294501	0	0	NFKBIZ
100521143	3.274	6.98	1.09781958	9.28E-48	1.73E-48	EPDR1
100521185	8.974	21.394	1.253044741	1.10E-108	1.04E-109	ZFAND2A
100521388	6.414	16.07	1.356552409	4.11E-146	2.90E-147	CRTAM
100521665	1.176	2.758	1.140019374	4.50E-09	2.94E-09	TPPP3
100521670	0.294	0.994	1.775358994	1.33E-11	7.33E-12	RASL11B
100521797	0.012	0.054	2.290570386	0.000824574	0.000916488	GRIN2D
100522293	56.424	116.666	1.029090523	0	0	CFP
100522469	83.006	209.796	1.293184118	0	0	DUSP1
100522635	3.152	15.894	2.164478885	2.24E-249	1.01E-250	GPR183
100522679	28.622	94.698	1.711274889	0	0	PPP1R15A
100522787	0.1	0.222	1.143221645	0.000427416	0.000455183	LOC100522787
100523046	0.486	1.688	1.732727788	9.30E-17	3.96E-17	C2H19orf24

Gene ID	F1 FPKM	F3 FPKM	log2(F3/F1)	Qvalue(F1-vs-F3)	Pvalue(F1-vs-F3)	Other Gene ID
100523358	24.584	53.208	1.10406166	0	0	JUNB
100524123	0.918	2.252	1.288553101	1.85E-16	7.96E-17	CXCR3
100524265	4.616	11.796	1.331563862	7.66E-50	1.38E-50	CXCL13
100524409	0.476	1.054	1.143571782	3.50E-15	1.60E-15	RBBP8NL
100524628	0.408	1.814	2.177802456	6.58E-16	2.90E-16	TNFRSF4
100524723	0.306	0.87	1.463485994	4.20E-07	3.24E-07	CHAC1
100525205	0.272	1.672	2.618992243	8.15E-26	2.53E-26	FOSL1
100525396	3.118	9.266	1.532160058	9.25E-49	1.70E-49	LOC100525396
100525703	9.114	21.118	1.023168095	3.15E-149	2.19E-150	DNAJB4
100621090	6.416	14.398	1.177467296	5.50E-80	6.91E-81	GADD45B
100621284	0.056	0.164	1.534495969	5.06E-08	3.59E-08	UNC5C
100621378	0.066	1.714	5.192154445	1.01E-80	1.26E-81	TREX2
100621540	0	0.992	7.189746017	1.95E-17	8.10E-18	LDHB
100621791	7.676	16.556	1.087107164	2.08E-56	3.40E-57	C14H10orf10
100621861	0.864	1.752	1.02753598	7.26E-09	4.82E-09	MNS1
100622156	12.736	37.384	1.474397895	0	0	TNFAIP3
100623310	0.156	0.532	1.815232377	3.01E-05	2.78E-05	RRAD
100623410	0.162	0.466	1.475566127	5.95E-07	4.64E-07	CCR4
100623564	0.08	0.224	1.55165652	0.000349888	0.00036836	TLE6
100623585	0.13	0.378	1.509768883	1.70E-11	9.45E-12	CR1
100624047	0.022	0.06	1.409425203	0.000136071	0.000135607	LRP1B
100624251	0.27	0.724	1.457887403	0.000138487	0.000138155	LOC100624251
100625944	2.65	7.746	1.48133164	3.73E-24	1.20E-24	RGCC
100626468	8.208	16.26	1.012923385	9.29E-163	5.92E-164	SLC2A3
100626906	1.66	3.4	1.020838502	3.06E-24	9.86E-25	SIX5
100626975	0.3	0.864	1.485877264	3.11E-12	1.65E-12	BHLHA15
100627130	0.032	0.254	3.099996866	0.000109701	0.000108182	CAMK2N2
100627312	0.176	0.44	1.271461563	1.44E-05	1.29E-05	NUMBL
100627600	0.71	1.548	1.145479909	1.61E-30	4.37E-31	GALNT5
100627879	0.488	1.414	1.442250332	1.05E-09	6.55E-10	LOC100627879
100627952	0.366	0.944	1.272940492	3.66E-08	2.57E-08	MS4A1
100628052	7.404	15.462	1.065760112	9.17E-59	1.44E-59	C17H8orf4
100736733	354.29	802.028	1.16325016	0	0	LOC100736733
100737113	0.334	1.034	1.502229695	4.98E-08	3.53E-08	LOC100737113

Gene ID	F1 FPKM	F3 FPKM	log2(F3/F1)	Qvalue(F1-vs-F3)	Pvalue(F1-vs-F3)	Other Gene ID
100737739	1.962	4.65	1.24522204	9.10E-26	2.82E-26	RND1
100738064	0.16	0.68	2.194645966	3.34E-06	2.79E-06	PLA2G2A
100738575	0.148	0.58	1.888580896	0.000136387	0.000135941	LOC100738575
100738612	39.488	147.994	1.87545044	0	0	ATF3
100738739	0.716	1.466	1.091418281	2.68E-12	1.42E-12	C2CD4B
100739001	0.058	0.286	2.290570386	0.000824574	0.000916488	NXNL2
100739218	335.552	724.694	1.098005126	0	0	LOC100739218
100739379	0.364	0.876	1.27044879	2.48E-06	2.05E-06	ASGR2
102161937	0.752	2.072	1.494769691	2.83E-15	1.29E-15	IER5L
102162743	2.986	6.446	1.102829879	3.81E-42	7.89E-43	FAM174B
102166057	0.09	0.272	1.471486172	0.000780908	0.000864901	RGS9
106504170	3.258	6.73	1.025125112	5.76E-46	1.11E-46	OTUD1
106504776	0.236	0.532	1.126707138	0.000778935	0.000862377	LOC106504776
106505000	1.298	2.922	1.143728998	5.86E-20	2.18E-20	LOC106505000
106505696	0.226	0.52	1.232974085	9.23E-11	5.37E-11	DZIP1L
110255204	0.62	1.776	1.678325131	2.84E-24	9.14E-25	LOC110255204
110255508	1.554	3.202	1.025298575	3.47E-24	1.12E-24	CER1
110255652	0.362	0.772	1.198350361	8.30E-06	7.23E-06	LOC110255652
110256018	0.308	0.62	1.003472276	1.54E-05	1.38E-05	EPOP
110256612	0.77	1.696	1.145212606	6.11E-11	3.52E-11	NEUROG3
110257437	1.88	3.768	1.005822599	4.09E-29	1.15E-29	TGIF2
110257549	0.536	1.102	1.063704488	3.75E-07	2.87E-07	C18H7orf57
110257782	0.744	1.662	1.255738659	5.99E-06	5.14E-06	LOC110257782
110258079	1.482	3.776	1.364570968	1.57E-26	4.74E-27	LRRC26
110258320	0.232	0.44	1.03892937	7.76E-05	7.53E-05	LOC110258320
110258711	0.13	0.592	2.181230597	1.58E-05	1.41E-05	LOC110258711
110258721	0.304	0.632	1.082076099	0.000613222	0.00066994	LOC110258721
110258824	0.112	0.716	2.618028415	4.70E-06	4.00E-06	LOC110258824
110259314	84.444	198.982	1.214188852	0	0	JUND
110260310	26.318	66.974	1.349087549	3.66E-289	1.44E-290	LOC110260310
110260624	0.11	0.548	2.312732036	7.92E-12	4.31E-12	TNFRSF13C
110260748	0.348	0.966	1.53683991	5.15E-06	4.39E-06	CLEC4E
110260749	0.048	0.366	3.037233436	5.66E-06	4.84E-06	LOC110260749
110260774	0.772	2.058	1.39161022	3.27E-25	1.03E-25	ENDOU

Gene ID	F1 FPKM	F3 FPKM	log2(F3/F1)	Qvalue(F1-vs-F3)	Pvalue(F1-vs-F3)	Other Gene ID
110260904	3.948	8.102	1.042002671	1.95E-91	2.13E-92	OSGIN1
110261005	0.23	0.466	1.042642873	4.01E-05	3.76E-05	NKPD1
110261009	23.232	126.616	2.421742888	0	0	FOSB
110261354	1.484	3.872	1.393678345	1.04E-06	8.32E-07	LOC110261354
110261743	0.15	0.642	2.037172002	1.54E-12	8.04E-13	LOC110261743
396562	0.092	0.26	1.502074491	0.000148927	0.000149173	GFAP
396583	0.248	0.688	1.417038388	5.45E-06	4.66E-06	SNAI1
396648	458.098	988.094	1.105099952	0	0	HSP70.2
396659	19.424	69.596	1.809579655	0	0	CXCR4
396663	0.102	1.404	3.776831382	1.36E-36	3.18E-37	CCR7
396668	23.806	48.24	1.028737271	1.03E-33	2.56E-34	CCL4
396741	199.842	535.994	1.408520608	0	0	DNAJB1
396769	1.376	2.856	1.067998447	2.26E-13	1.13E-13	MMP3
396862	9.926	22.318	1.112721309	1.19E-42	2.45E-43	TIMP1
396880	62.394	148.716	1.204941375	0	0	CXCL8
396913	206.76	532.814	1.362100131	0	0	JUN
396915	2.15	6.078	1.449723648	6.58E-46	1.27E-46	EDN1
396948	5.834	11.892	1.024794871	7.17E-39	1.59E-39	HSD11B2
397106	0.892	3.522	1.979541124	1.02E-31	2.66E-32	IL10
397122	2.506	6.264	1.342289936	3.99E-41	8.46E-42	IL1B
397165	15.376	41	1.411580228	4.54E-106	4.38E-107	CD69
397195	17.758	38.002	1.081117021	2.27E-143	1.63E-144	ADM
397208	0.298	0.896	1.659314233	3.30E-05	3.07E-05	CSF2
397210	0.168	0.35	1.115399215	0.000370976	0.000391637	TRPC3
397231	1.634	3.286	1.005168167	3.57E-07	2.73E-07	CD40LG
397251	4.216	17.226	2.058305274	0	0	PLK2
397278	22.302	48.61	1.12898787	6.39E-141	4.67E-142	PMAIP1
397286	1.328	4.662	1.799371722	2.22E-19	8.47E-20	CTLA4
397316	21.714	48.608	1.154198052	4.63E-151	3.19E-152	FCN1
397382	1.004	2.412	1.263721962	2.48E-21	8.78E-22	MUC1
397400	0.118	0.374	1.606543758	9.26E-07	7.34E-07	TGFB3
397407	1.47	3.086	1.058584417	3.56E-05	3.31E-05	CLPS
397423	0.842	7.336	3.258580272	8.26E-42	1.73E-42	FABP6
397479	0.936	3.768	2.031036253	7.39E-135	5.68E-136	NR4A3

Gene ID	F1 FPKM	F3 FPKM	log2(F3/F1)	Qvalue(F1-vs-F3)	Pvalue(F1-vs-F3)	Other Gene ID
397544	0.572	2.842	2.345419867	3.03E-59	4.74E-60	RGS16
397564	5.554	12.534	1.169128072	2.56E-95	2.71E-96	HBEGF
397590	0.302	0.684	1.187188579	1.17E-08	7.90E-09	PTGS2
397612	0.14	0.526	1.890639779	5.67E-05	5.42E-05	LY49
397613	0.196	0.562	1.612632867	7.12E-10	4.39E-10	DNAJA4
397669	0.176	0.542	1.606543758	9.26E-07	7.34E-07	CD19
403123	0.152	1.474	3.400194877	3.78E-09	2.46E-09	IL21
404704	1.11	2.23	1.024495526	8.96E-17	3.80E-17	CD4
414904	0.64	4.086	2.662443166	1.47E-37	3.36E-38	CXCL2
444998	0.24	0.496	1.457910905	1.64E-08	1.12E-08	FOXP3
445518	12.548	26.18	1.056491738	1.81E-52	3.14E-53	SPAI-2
448981	35.966	75.832	1.072141733	0	0	C2
449530	0.054	0.422	2.797530375	1.12E-05	9.85E-06	IL17A
492313	2.536	8.266	1.669053236	2.09E-208	1.12E-209	THBS1
574055	0.098	0.532	2.502074491	0.000171076	0.000172475	TNFSF8
595104	1.606	7.548	2.146665937	1.39E-54	2.34E-55	IL22
595111	38.188	85.662	1.161308857	0	0	KLF4
733597	2.412	7.196	1.49556706	3.29E-79	4.18E-80	ICOS
733613	0.63	1.602	1.31101542	2.26E-16	9.78E-17	IL23R
780438	1.03	2.132	1.042642873	9.11E-06	7.97E-06	SMPX
780439	26.792	85.516	1.667526407	0	0	SOD3
