# Supplementary material for: Transcriptome Profile Analysis of Intestinal Upper Villus Epithelial Cells and Crypt Epithelial Cells of Suckling Piglets
Source: Animals (Basel). 2022 Sep 7;12(18):2324. doi: 10.3390/ani12182324 (PMC9494997; doi:10.3390/ani12182324)
Supplement: Supplementary file 1 [file animals-12-02324-s001.zip › Table S2.The down-regulation DEGs of F3 compared with F1.pdf]

Figure S2. The down-regulation DEGs of the crypt cells (F3) compared with the villus upper cells (F1)

Gene ID	F1 FPKM	F3 FPKM	log2(F3/F1)	Qvalue(F1-vs-F3)	Pvalue(F1-vs-F3)	Other Gene ID
100037275	1.122	0.482	-1.3293259	5.26E-14	2.55E-14	SPARCL1
100037946	0.284	0.096	-1.5169869	2.43E-10	1.46E-10	KCND3
100037954	0.488	0.23	-1.1552965	5.08E-05	4.83E-05	C8B
100037955	2.694	0.502	-2.4066645	9.97E-20	3.74E-20	C8G
100101551	0.216	0.094	-1.2955259	8.34E-09	5.56E-09	COL6A3
100125375	0.468	0.148	-1.6287344	4.24E-08	2.99E-08	CAV2
100125829	554.3	267.896	-1.0495586	0	0	MUC13
100125974	2.53	0.844	-1.5937688	1.80E-23	5.97E-24	SPO11
100126276	0.438	0.296	-1.0153045	3.61E-06	3.03E-06	WNT10B
100126281	116.116	40.12	-1.5261663	0	0	CYP3A46
100126282	3.116	1.058	-1.5423196	1.77E-35	4.21E-36	CYP27A1
100126283	4.558	1.44	-1.570883	1.45E-41	3.04E-42	AQP7
100126284	0.16	0.03	-2.0277465	7.84E-06	6.81E-06	PTGIS
100126844	16.916	6.628	-1.3664372	3.99E-131	3.17E-132	GATM
100134959	48.164	19.194	-1.3092187	8.65E-173	5.30E-174	G6PC
100144303	147.07	72.556	-1.0042316	0	0	ACE2
100144468	2.674	1.292	-1.0446979	1.62E-13	8.05E-14	CYP3A22
100144531	66.816	24.626	-1.4340834	0	0	PCK1
100144588	10.344	3.936	-1.385105	5.17E-102	5.17E-103	SLC3A1
100144620	27.048	10.408	-1.3863581	4.11E-130	3.28E-131	AQP10
100151780	31.592	9.256	-1.7596044	0	0	KCNJ13
100151853	1.244	0.362	-1.778387	1.01E-10	5.91E-11	C15H2orf66
100152001	3.826	1.994	-1.030553	1.64E-44	3.24E-45	COL3A1
100152015	0.996	0.432	-1.1108316	3.86E-06	3.25E-06	HMX3
100152067	1.588	0.54	-1.5153526	6.35E-31	1.70E-31	ADCY4
100152112	6.028	2.45	-1.3245998	4.89E-224	2.43E-225	ABCA1
100152205	36.69	16.12	-1.1827538	0	0	DST
100152401	3.094	0.928	-1.8670905	8.26E-61	1.27E-61	POSTN
100152607	1.124	0.44	-1.4913357	6.82E-11	3.93E-11	LUM
100152930	2.022	0.998	-1.0178157	5.78E-25	1.83E-25	ADGRF5
100152957	0.326	0.096	-1.6720269	2.14E-06	1.75E-06	EPB42
100153434	32.684	15.668	-1.0604176	0	0	ABHD2
100153454	1.158	0.614	-1.0247851	5.88E-18	2.39E-18	COL4A2

Gene ID	F1 FPKM	F3 FPKM	log2(F3/F1)	Qvalue(F1-vs-F3)	Pvalue(F1-vs-F3)	Other Gene ID
100153508	0.06	0.014	-2.3496745	0.000457299	0.000489061	NPR1
100153645	3.892	1.604	-1.2303756	1.01E-11	5.52E-12	CALB1
100153739	0.092	0.02	-2.0277465	7.84E-06	6.81E-06	CCDC13
100153854	1.008	0.236	-2.0650214	1.60E-12	8.36E-13	LOC100153854
100153917	0.172	0.06	-1.5423196	7.14E-05	6.90E-05	SLITRK6
100153974	0.082	0.04	-1.1921484	0.000815964	0.000905855	CTNNA3
100154297	0.712	0.272	-1.4189133	2.60E-08	1.81E-08	AMHR2
100154315	0.084	0.008	-3.44418	7.13E-05	6.89E-05	DCST2
100154502	320.22	134.38	-1.2589029	0	0	PRAP1
100154581	0.314	0.144	-1.1881373	5.79E-07	4.52E-07	LAMA4
100154587	0.072	0.01	-2.9573571	0.000523057	0.000565448	KCNMB4
100154624	482.936	204.33	-1.2669212	0	0	CLCA4
100154649	0.276	0.084	-1.4373019	0.00017618	0.000177978	RAI2
100154895	0.286	0.132	-1.0898074	0.000826277	0.000918621	TAL2
100154962	2.94	0.966	-1.5840686	1.15E-45	2.21E-46	PTPRR
100155042	0.324	0.138	-1.2212078	3.67E-05	3.42E-05	BOLL
100155204	2.834	0.89	-1.6619012	2.04E-36	4.75E-37	ZBBX
100155308	0.164	0.008	-4.2231213	6.33E-06	5.45E-06	LOC100155308
100155458	0.22	0.096	-1.1800113	8.35E-06	7.27E-06	CLVS2
100155607	0.718	0.278	-1.3250889	1.53E-10	9.05E-11	MATN2
100155660	0.32	0.156	-1.2695383	5.13E-05	4.88E-05	CD93
100155871	1.612	0.226	-2.8705042	1.57E-70	2.16E-71	SLC16A9
100156014	1.336	0.572	-1.2197747	7.38E-10	4.56E-10	IL17RB
100156181	0.364	0.088	-2.1162447	9.07E-33	2.30E-33	PTPRB
100156333	0.198	0.042	-2.0803479	0.000282794	0.000294119	LOC100156333
100156404	1.688	0.748	-1.1434534	1.58E-08	1.08E-08	PGF
100156419	1.332	0.516	-1.3883244	7.38E-57	1.19E-57	HOMER2
100156461	14.518	3.912	-1.9152931	0	0	BMP1
100156478	0.028	0.01	-1.764712	0.000740985	0.000818859	DNAH8
100156646	1.244	0.622	-1.0199851	6.60E-25	2.09E-25	SVOP
100157065	11.658	3.964	-1.5595964	0	0	ASAH2
100157073	0.456	0.196	-1.1876547	0.000232704	0.00023909	AGT
100157235	0.362	0.098	-1.7585158	2.97E-06	2.47E-06	ITIH3
100157319	7.938	2.508	-1.7346172	1.56E-51	2.75E-52	SDR16C5

Gene ID	F1 FPKM	F3 FPKM	log2(F3/F1)	Qvalue(F1-vs-F3)	Pvalue(F1-vs-F3)	Other Gene ID
100157330	0.22	0.108	-1.0206953	7.84E-09	5.23E-09	CLSTN2
100157365	0.426	0.162	-1.495777	1.30E-05	1.15E-05	CLEC14A
100157523	13.198	4.928	-1.4468592	2.24E-155	1.48E-156	GGT5
100157775	5.476	2.52	-1.113228	6.83E-145	4.87E-146	TRPM6
100157784	0.252	0.126	-1.0552522	5.83E-08	4.15E-08	SV2B
100158056	21.044	10.026	-1.027092	9.11E-173	5.60E-174	PFKFB4
100158128	0.114	0.008	-3.8555655	0.000200765	0.000204355	LMAN1L
100158166	2.25	1.074	-1.122168	7.92E-17	3.36E-17	RFLNA
100158187	0.268	0.126	-1.1617156	0.00069663	0.000766512	PLS3
100169744	2.958	1.272	-1.2079006	1.26E-11	6.95E-12	CXCL11
100170651	6.998	1.192	-2.6023444	2.65E-91	2.90E-92	SLC30A2
100233169	180.446	69.004	-1.360389	0	0	ACSL3
100233182	22.96	8.984	-1.188358	3.48E-237	1.62E-238	GK
100270723	1.38	0.208	-2.6418553	6.81E-08	4.89E-08	RBP7
100271745	1.562	0.61	-1.2631656	3.80E-06	3.19E-06	TCAP
100286744	4.344	1.518	-1.4964431	1.07E-43	2.15E-44	MUC12
100294675	32.452	14.162	-1.2029343	1.15E-146	8.13E-148	DGAT2
100294688	397.29	190.422	-1.0866095	0	0	UBD
100337678	0.666	0.228	-1.4907893	3.50E-05	3.26E-05	PNCK
100381252	3.416	1.716	-1.1126354	1.12E-05	9.90E-06	APOC2
100510957	0.31	0.156	-1.0217901	0.00071193	0.000784378	ACSM2B
100511263	0.824	0.306	-1.4565898	4.31E-11	2.46E-11	KCNC3
100511353	0.318	0.164	-1.0207825	2.01E-08	1.38E-08	ADAMTS12
100511431	0.04	0.008	-2.4744156	0.000730302	0.000806207	CRB2
100511434	2.406	1.07	-1.2688182	2.18E-12	1.15E-12	EGFL7
100511536	189.398	75.144	-1.3380311	0	0	MME
100511582	0.338	0.122	-1.4098693	5.99E-08	4.27E-08	SYT2
100511633	1.076	0.454	-1.2248423	5.29E-07	4.11E-07	RDH16
100511642	13.712	4.844	-1.478369	0	0	LAMA3
100511654	0.076	0.006	-3.6577968	0.000607619	0.000663114	SERTAD4
100511672	0.124	0.02	-2.6328257	0.00084381	0.00093897	SLC24A5
100511847	0.138	0.054	-1.4318653	0.000121776	0.000120796	CPED1
100511870	0.59	0.228	-1.3052804	0.000150039	0.000150374	GNG13
100512046	0.584	0.154	-2.0162508	3.23E-09	2.09E-09	EFEMP1

Gene ID	F1 FPKM	F3 FPKM	log2(F3/F1)	Qvalue(F1-vs-F3)	Pvalue(F1-vs-F3)	Other Gene ID
100512361	0.492	0.124	-2.0966319	3.51E-19	1.35E-19	PPFIA4
100512640	0.81	0.23	-1.8902429	2.15E-21	7.62E-22	CCDC129
100512657	0.334	0.1	-1.7349647	1.66E-05	1.49E-05	WIF1
100512756	0.568	0.194	-1.5828402	5.31E-09	3.49E-09	MEIS2
100512772	0.156	0.03	-2.2792852	0.000184846	0.000187268	TMEM270
100512834	0.076	0.024	-1.6678505	0.000256814	0.000265499	KIRREL
100513233	0.096	0	-5.1192448	1.59E-05	1.42E-05	LOC100513233
100513377	0.242	0.108	-1.1228933	2.85E-07	2.16E-07	IL17RD
100513444	0.212	0.066	-1.7238125	2.23E-10	1.34E-10	SLIT3
100513689	4.194	1.826	-1.2009723	6.70E-85	7.76E-86	NCKAP5
100513769	0.182	0.06	-1.6802323	7.35E-09	4.89E-09	TAS2R16
100513818	1.91	0.682	-1.496026	2.85E-20	1.05E-20	ZMYND15
100513844	1	0.438	-1.1555752	6.43E-06	5.54E-06	LOC100513844
100513853	0.35	0.15	-1.2274463	1.17E-07	8.59E-08	ANTXR1
100513942	0.258	0.052	-2.1100147	0.000398899	0.000422964	LOC100513942
100514201	5.672	1.926	-1.519659	6.48E-81	8.04E-82	FRMD1
100514396	0.576	0.28	-1.1272821	8.94E-10	5.56E-10	NYAP1
100514537	1.604	0.72	-1.181117	2.65E-24	8.53E-25	KCNH6
100514576	4.88	2.468	-1.009867	8.98E-48	1.67E-48	ADAMTSL4
100514726	41.008	10.986	-1.8920591	0	0	ENPP7
100514743	4.186	1.494	-1.4904686	7.24E-124	6.07E-125	WIPF3
100514914	0.482	0.084	-2.4846041	1.54E-14	7.22E-15	CATSPERE
100514979	1.812	0.658	-1.4565125	5.59E-25	1.77E-25	LOC100514979
100515047	0.344	0.14	-1.1208949	1.29E-06	1.03E-06	MAGI2
100515049	2.294	0.604	-1.9214376	7.71E-71	1.06E-71	LOC100515049
100515143	1.088	0.448	-1.2748466	5.24E-26	1.61E-26	RNF207
100515194	0.406	0.162	-1.2172243	5.56E-06	4.76E-06	ABLIM3
100515310	3.132	1.508	-1.0392013	1.65E-38	3.69E-39	WDR97
100515322	0.236	0.082	-1.4598575	6.29E-06	5.41E-06	CDH2
100515336	2.228	1.166	-1.0446164	6.63E-36	1.56E-36	COL4A1
100515394	1.15	0.442	-1.4111101	2.06E-10	1.23E-10	LOC100515394
100515404	0.308	0.132	-1.3149091	4.68E-06	3.97E-06	ITIH5
100515407	0.1	0.02	-2.237465	1.83E-05	1.65E-05	HSF5
100515575	0.628	0.276	-1.1617156	0.00069663	0.000766512	4-Sep

Gene ID	F1 FPKM	F3 FPKM	log2(F3/F1)	Qvalue(F1-vs-F3)	Pvalue(F1-vs-F3)	Other Gene ID
100515579	2.698	0	-11.160399	8.04E-159	5.22E-160	LOC100515579
100515668	0.606	0.286	-1.0571762	1.63E-07	1.21E-07	BMP8A
100515715	0.202	0.046	-2.0898074	1.24E-07	9.07E-08	PDE6A
100515788	3.914	1.446	-1.450314	9.70E-08	7.07E-08	LOC100515788
100515852	0.494	0.22	-1.1166527	2.55E-05	2.34E-05	LOC100515852
100515955	0.54	0.172	-1.6163202	3.37E-15	1.54E-15	PTPRU
100515970	1.694	0.744	-1.1854317	1.14E-21	4.00E-22	OSBP2
100516141	120.128	47.734	-1.3258841	0	0	MEP1B
100516302	0.27	0	-4.4167887	0.000823765	0.000915232	LOC100516302
100516366	0.326	0.12	-1.581848	0.000353173	0.000372075	DPT
100516653	0.254	0.1	-1.2893494	2.75E-09	1.77E-09	CCDC170
100516697	0.552	0.258	-1.1061851	2.12E-26	6.45E-27	KALRN
100516922	0.302	0.154	-1.1856261	2.30E-05	2.10E-05	MMRN1
100517042	0.114	0.038	-1.5176576	7.66E-05	7.43E-05	GABRG1
100517148	1.5	0.462	-1.6611559	1.59E-18	6.29E-19	SCGB1C1
100517588	0.598	0.284	-1.1939781	7.62E-11	4.41E-11	DNAAF1
100517626	1.278	0.608	-1.0202126	2.90E-06	2.41E-06	ARL13A
100517800	0.724	0.316	-1.1163418	2.02E-06	1.65E-06	LOC100517800
100518179	0.164	0.064	-1.49341	2.13E-05	1.94E-05	FLT4
100518683	0.158	0.026	-2.5423196	4.79E-08	3.40E-08	PRG4
100518817	0.478	0.186	-1.4230207	4.03E-05	3.78E-05	ALAS2
100519011	26.03	11.486	-1.1901142	0	0	SLC5A12
100519341	0.858	0.39	-1.0959034	1.64E-07	1.22E-07	IDO2
100519488	5.65	0.744	-2.8446693	3.48E-211	1.82E-212	MOV10L1
100519519	0.026	0.01	-1.1479956	2.18E-08	1.50E-08	TTN
100519539	3.044	1.064	-1.5479136	3.12E-94	3.32E-95	KLHL3
100519570	0.22	0.108	-1.0868844	0.000483709	0.000519548	KLLN
100519724	1.138	0.506	-1.1676283	9.47E-15	4.41E-15	MAP6
100520061	0.06	0.014	-2.2792852	0.000184846	0.000187268	ATP2B3
100520186	7.164	3.014	-1.1858209	9.47E-32	2.47E-32	SH2D6
100520235	0.936	0.27	-1.6735642	5.36E-07	4.17E-07	PDLIM4
100520273	75.136	14.02	-2.4124389	0	0	LOC100520273
100520329	0.11	0.03	-2.0263885	0.000257073	0.000265803	LOC100520329
100520648	0.278	0.018	-4.04482	1.82E-08	1.25E-08	SLC6A18

Gene ID	F1 FPKM	F3 FPKM	log2(F3/F1)	Qvalue(F1-vs-F3)	Pvalue(F1-vs-F3)	Other Gene ID
100520667	1.882	0.868	-1.1651454	2.15E-09	1.38E-09	ACTG2
100520976	0.8	0.366	-1.0654151	3.37E-16	1.47E-16	SLC13A5
100521229	3.436	1.578	-1.063002	3.29E-20	1.21E-20	LOC100521229
100521401	7.016	2.746	-1.3231296	5.35E-27	1.60E-27	TMIGD1
100521529	0.218	0.104	-1.0738429	0.000236699	0.000243435	LOC100521529
100521710	3.996	1.896	-1.0678309	1.61E-73	2.15E-74	NAIF1
100521795	1.908	0.786	-1.0004258	7.58E-09	5.04E-09	SYNE4
100521917	0.108	0.054	-1.09547	1.24E-05	1.10E-05	PTPN14
100521955	0.182	0.064	-1.3729877	0.000724093	0.000798828	MEIKIN
100522021	30.738	15.026	-1.0361556	0	0	LRRC66
100522035	0.552	0.24	-1.2250104	2.02E-06	1.65E-06	TM4SF1
100522166	31.24	10.354	-1.5977288	0	0	TMEM37
100522231	33.148	15.518	-1.0826378	4.56E-218	2.30E-219	SLC28A2
100522267	0.272	0.114	-1.3138985	1.33E-05	1.18E-05	LOC100522267
100522389	0.642	0.316	-1.1470559	3.50E-05	3.26E-05	CCDC153
100522737	0.104	0.01	-3.6577968	0.000607619	0.000663114	ANKRD33
100522753	0.498	0.19	-1.3411798	1.74E-23	5.76E-24	DNAH14
100522927	8.898	4.368	-1.0408571	4.52E-33	1.14E-33	CGREF1
100523091	9.652	4.256	-1.1567132	1.03E-103	1.01E-104	CDRT1
100523107	0.616	0.266	-1.3071643	2.87E-07	2.18E-07	LOC100523107
100523209	10.194	3.31	-1.5894935	0	0	COL7A1
100523256	0.078	0.014	-2.7450832	1.51E-07	1.11E-07	LOC100523256
100523279	0.31	0.132	-1.1853227	5.44E-06	4.65E-06	CDRT4
100523323	0.16	0.058	-1.2392641	0.000257864	0.000266697	NOX4
100523371	319.742	127.488	-1.3452616	0	0	APOB
100523492	47.344	21.732	-1.1282986	6.49E-279	2.59E-280	LOC100523492
100523637	0.94	0.446	-1.0323192	1.75E-07	1.30E-07	KCNAB3
100523915	0.096	0.036	-1.442784	0.000374481	0.000395608	SEMA3D
100524003	1.074	0.424	-1.3374379	3.51E-07	2.68E-07	TAS2R41
100524016	0.422	0.024	-4.2052846	1.82E-09	1.16E-09	LOC100524016
100524201	4.722	1.772	-1.4403478	2.95E-67	4.19E-68	FUT2A
100524266	33.904	10.7	-1.6701564	0	0	GPAT3
100524361	0.12	0.03	-1.8922621	1.21E-07	8.91E-08	QRFP
100524377	8.834	3.192	-1.4891471	1.27E-81	1.55E-82	NTN5

Gene ID	F1 FPKM	F3 FPKM	log2(F3/F1)	Qvalue(F1-vs-F3)	Pvalue(F1-vs-F3)	Other Gene ID
100524382	0.356	0.156	-1.1546759	2.16E-05	1.97E-05	LOC100524382
100524435	0.114	0.034	-1.764712	0.000740985	0.000818859	SLC16A8
100524623	2.608	0.662	-1.8877517	2.18E-26	6.65E-27	TMEM117
100524697	0.678	0.338	-1.0166763	3.94E-09	2.57E-09	OMP
100524750	45.54	19.034	-1.2840045	1.78E-301	6.54E-303	CYP2J34
100524811	1.2	0.494	-1.2518993	2.28E-16	9.85E-17	SCNN1D
100524821	0.146	0.036	-2.04482	4.95E-05	4.69E-05	ROBO4
100525112	32.86	11.33	-1.5495238	1.87E-298	7.05E-300	LOC100525112
100525120	0.072	0.016	-2.0186573	0.000499088	0.000537585	HGF
100525121	0.59	0.284	-1.0574686	1.18E-15	5.28E-16	NAV1
100525134	0.182	0.074	-1.442784	4.55E-05	4.29E-05	KIAA1456
100525140	2.866	1.25	-1.1775269	1.74E-52	3.03E-53	LOC100525140
100525146	5.284	2.552	-1.0346875	1.05E-83	1.25E-84	PLXNB3
100525263	27.658	11.012	-1.3361164	3.83E-270	1.60E-271	FAXDC2
100525278	0.144	0.01	-3.6577968	0.000607619	0.000663114	LOX
100525324	1.536	0.492	-1.5877115	4.34E-35	1.04E-35	L1CAM
100525348	0.242	0.078	-1.7767849	1.08E-06	8.58E-07	ADGRL4
100525453	1.402	0.596	-1.1714819	9.71E-18	3.98E-18	DPYSL3
100525565	0.136	0.062	-1.0924676	0.000161859	0.000162736	FSTL4
100525607	0.914	0.286	-1.5938499	3.00E-14	1.43E-14	NGFR
100525613	6.344	1.694	-1.8714054	7.89E-11	4.58E-11	LOC100525613
100525676	0.254	0.098	-1.3463994	0.000120494	0.000119437	PDZD4
100525762	0.494	0.166	-1.5614285	5.58E-07	4.35E-07	DDIT4L
100526058	1.162	0.454	-1.3415337	3.84E-27	1.14E-27	SLFNL1
100526221	0.18	0.078	-1.1717307	0.000372773	0.00039375	SLC17A8
100526237	0.282	0.076	-1.9313619	7.80E-07	6.14E-07	FMOD
100533558	2.732	0.826	-1.6566754	2.41E-52	4.21E-53	TRPM5
100579172	0.38	0.178	-1.0104685	1.43E-07	1.06E-07	APC2
100620258	0.432	0.086	-2.3684851	3.11E-11	1.76E-11	THPO
100620350	1.968	0.792	-1.2694468	7.42E-25	2.36E-25	CHRD
100620374	6.38	1.938	-1.7301828	4.38E-94	4.68E-95	BMP8B
100620394	0.432	0.202	-1.1864911	2.74E-08	1.91E-08	COL15A1
100620540	0.588	0.244	-1.2501389	1.54E-06	1.24E-06	LOC100620540
100620587	0.296	0.116	-1.5423196	0.000318663	0.000334195	IRX2

Gene ID	F1 FPKM	F3 FPKM	log2(F3/F1)	Qvalue(F1-vs-F3)	Pvalue(F1-vs-F3)	Other Gene ID
100621059	0.276	0.078	-2.0407731	8.26E-11	4.80E-11	ADGRF2
100621352	2.168	1.082	-1.0129495	3.27E-12	1.74E-12	LOC100621352
100621607	1.45	0.402	-1.7247863	9.54E-16	4.24E-16	LOC100621607
100621671	115.454	39.018	-1.5883739	0	0	ENPP3
100621753	0.812	0.28	-1.5005439	4.40E-09	2.87E-09	PHOSPHO1
100622304	0.578	0.164	-1.7913472	1.07E-08	7.20E-09	DCST1
100622305	0.308	0.148	-1.0511488	0.000425958	0.000453507	PTGIR
100622520	0.618	0.304	-1.2017554	1.11E-05	9.76E-06	GNG8
100622802	0.226	0.09	-1.2979791	2.83E-05	2.61E-05	CXHXorf36
100623173	0.086	0.03	-1.5423196	0.000193895	0.000197138	ESPNL
100623257	1.174	0.558	-1.0421359	1.56E-10	9.26E-11	LOC100623257
100623339	0.308	0.032	-3.1272821	5.00E-06	4.25E-06	QPRT
100623602	0.064	0.01	-2.4979255	4.65E-10	2.85E-10	HMCN2
100624186	4.768	1.682	-1.4239249	5.66E-40	1.22E-40	GPRC5A
100624435	4.782	1.152	-2.0722797	5.40E-74	7.18E-75	LOC100624435
100624460	0.048	0.006	-2.9177326	1.88E-10	1.12E-10	LOC100624460
100624918	1.246	0.33	-1.8631474	1.07E-09	6.67E-10	LOC100624918
100624981	0.572	0.142	-1.6109598	1.01E-07	7.33E-08	12-Sep
100625267	0.222	0.074	-1.572067	4.81E-05	4.55E-05	SYT12
100625364	0.254	0.128	-1.2203915	0.000784639	0.000869488	ERG
100625920	25.212	8.896	-1.4527592	1.52E-149	1.05E-150	ADA
100625942	58.27	29.018	-1.0062372	1.61E-305	5.85E-307	BST1
100626199	0.876	0.324	-1.6447291	1.52E-11	8.42E-12	LOC100626199
100626716	1.68	0.714	-1.3207051	2.17E-31	5.75E-32	COL1A2
100626898	0.228	0.102	-1.1336818	3.31E-08	2.32E-08	LANCL3
100627072	3.258	1.372	-1.2890398	1.62E-102	1.61E-103	PTPRD
100627510	0.06	0.01	-2.6203221	0.000393543	0.000417057	CDH6
100627513	119.598	54.876	-1.1116178	0	0	STOM
100627924	0.538	0.278	-1.0114891	7.03E-10	4.34E-10	MYH11
100627929	11.012	4.838	-1.2883037	4.66E-41	9.89E-42	FLYWCH2
100627950	0.28	0.108	-1.2749585	1.22E-05	1.09E-05	ADAMTS13
100627962	1.322	0.64	-1.0323557	1.11E-06	8.83E-07	C4H1orf162
100628050	0.272	0.056	-2.3947624	8.88E-10	5.53E-10	MYBPC3
100628053	0.652	0.328	-1.0302331	2.35E-06	1.93E-06	FBXL2

Gene ID	F1 FPKM	F3 FPKM	log2(F3/F1)	Qvalue(F1-vs-F3)	Pvalue(F1-vs-F3)	Other Gene ID
100628113	6.106	2.97	-1.0252387	1.61E-122	1.37E-123	PHLPP2
100628129	0.344	0.058	-2.6852776	3.59E-09	2.33E-09	EMCN
100736591	0.31	0.144	-1.1280834	0.00081633	0.00090638	LYPD3
100736592	1.336	0.608	-1.1797495	9.53E-09	6.39E-09	FBXO44
100737235	0.894	0.442	-1.0255286	8.15E-09	5.44E-09	LRRC27
100737468	16.628	7.584	-1.1390626	2.47E-81	3.05E-82	IL34
100737517	156.626	71.798	-1.143821	0	0	SLC40A1
100737524	0.276	0.126	-1.1310721	1.14E-05	1.01E-05	PSD2
100737755	6.808	1.386	-2.2618456	3.40E-142	2.47E-143	LOC100737755
100737768	23.022	9.73	-1.2945925	3.22E-44	6.39E-45	LOC100737768
100737904	0.812	0.326	-1.2276893	0.000293772	0.000306387	LOC100737904
100738062	2.846	0.73	-2.0043206	4.30E-88	4.85E-89	ACOT12
100738075	0.808	0.402	-1.0170269	0.00014757	0.000147686	LOC100738075
100738360	1.94	0.93	-1.0740807	2.60E-09	1.67E-09	PDIA2
100739083	0.284	0.116	-1.2513729	2.54E-07	1.91E-07	FAM205A
100739158	0.358	0.122	-1.5289732	8.65E-15	4.02E-15	SUCNR1
100739471	0.152	0.048	-1.5896253	0.000961985	0.001077719	RFTN2
100739528	0.144	0.01	-3.7986594	5.69E-10	3.50E-10	LOC100739528
100739561	19.032	8.898	-1.1217288	2.91E-123	2.46E-124	LOC100739561
100739839	0.632	0.172	-1.8247194	1.12E-13	5.52E-14	SPATC1
102160115	59.062	23.358	-1.3545422	0	0	MGAM
102161210	4.418	2.07	-1.0634521	2.38E-18	9.55E-19	LOC102161210
102162017	0.408	0.182	-1.0799764	5.30E-07	4.12E-07	EFCAB8
102162205	0.314	0.098	-1.608004	1.29E-17	5.34E-18	LOC102162205
102162296	0.668	0.162	-2.0029645	3.09E-09	2.00E-09	LOC102162296
102162410	0.156	0.074	-1.1222985	0.000724662	0.000799561	GPR17
102162779	0.796	0.37	-1.0713209	0.000134609	0.000134052	C13H3orf35
102163220	0.39	0.19	-1.0553721	9.61E-09	6.45E-09	ASPRV1
102163819	114.19	53.91	-1.0719584	0	0	MS4A10
102164386	1.128	0.54	-1.0653994	4.19E-08	2.95E-08	HCAR2
102165015	8.356	2.424	-1.8733159	6.68E-105	6.50E-106	LOC102165015
102165270	0.434	0.192	-1.1676013	6.30E-07	4.92E-07	ZNF750
102165510	0.648	0.2	-1.635429	2.00E-06	1.63E-06	LOC102165510
102166010	0.442	0.216	-1.0718098	1.21E-07	8.89E-08	IQCH

Gene ID	F1 FPKM	F3 FPKM	log2(F3/F1)	Qvalue(F1-vs-F3)	Pvalue(F1-vs-F3)	Other Gene ID
102166052	0.194	0.054	-1.7913327	2.89E-05	2.67E-05	DPCR1
102166693	0.038	0.014	-1.6492348	9.50E-05	9.32E-05	HMCN1
102166764	0.322	0.11	-1.6761754	8.17E-08	5.91E-08	COLEC12
102167522	7.062	3.158	-1.0116667	6.71E-06	5.80E-06	LOC102167522
102167930	0.094	0.014	-3.0172629	0.000908472	0.001014609	LOC102167930
102168098	1.77	0.64	-1.3999667	1.85E-26	5.61E-27	LOC102168098
106504234	44.512	18.732	-1.2710059	0	0	LOC106504234
106504796	0.61	0.216	-1.5329055	5.54E-37	1.28E-37	LOC106504796
106505070	5.9	1.98	-1.5192171	5.90E-80	7.42E-81	RGS13
106505208	1.852	0.574	-1.7268166	1.21E-16	5.18E-17	LOC106505208
106506828	1.058	0.312	-1.7449488	8.52E-14	4.17E-14	LOC106506828
106507601	0.234	0.05	-2.2742269	0.000781526	0.000865699	LOC106507601
106508195	0.492	0.15	-1.6353251	6.31E-08	4.51E-08	TAS2R40
106508237	1.416	0.55	-1.352503	7.88E-06	6.85E-06	LOC106508237
106508546	0.32	0.118	-1.5313632	2.49E-09	1.60E-09	LOC106508546
106509227	0.404	0.134	-1.6954764	7.40E-05	7.16E-05	LOC106509227
106509580	1.062	0.458	-1.2470067	7.88E-08	5.70E-08	LOC106509580
106509652	3.304	1.338	-1.2883683	1.91E-14	9.06E-15	LOC106509652
106510141	1.026	0.47	-1.0808546	6.01E-05	5.77E-05	LOC106510141
106510284	44.022	20.158	-1.0791233	1.03E-239	4.74E-241	LOC106510284
110255181	0.47	0.124	-1.9304001	1.09E-06	8.70E-07	LOC110255181
110255271	0.568	0.152	-1.7228919	1.02E-05	8.94E-06	LOC110255271
110255553	2.548	1.112	-1.184258	3.88E-12	2.08E-12	LOC110255553
110255930	0.44	0.11	-2.084469	2.02E-10	1.21E-10	NOTUM
110255970	2.142	0.764	-1.4712521	2.01E-17	8.36E-18	LOC110255970
110256043	2.098	0.804	-1.5198669	4.74E-16	2.08E-16	LOC110256043
110256114	0.884	0.394	-1.1797495	4.29E-06	3.63E-06	PMP22
110256120	0.248	0.068	-1.9114872	0.000459389	0.000491496	MFAP4
110256150	1.86	0.622	-1.570713	2.10E-47	3.94E-48	KCNJ12
110256417	1.066	0.456	-1.2910145	6.38E-15	2.95E-15	FSTL1
110256522	0.284	0.104	-1.4891353	0.000511705	0.000552065	STKLD1
110256864	0.212	0.03	-2.764712	0.000135118	0.000134618	LOC110256864
110257013	0.33	0	-5.2742269	5.12E-06	4.37E-06	LOC110257013
110257397	7.638	3.606	-1.0626211	2.45E-78	3.13E-79	PTK6

Gene ID	F1 FPKM	F3 FPKM	log2(F3/F1)	Qvalue(F1-vs-F3)	Pvalue(F1-vs-F3)	Other Gene ID
110257399	1.456	0.53	-1.453783	8.81E-12	4.81E-12	SRMS
110257482	0.184	0.078	-1.2067166	1.22E-07	8.95E-08	LAMA5
110257613	0.27	0.092	-1.507906	0.000285349	0.000296942	LOC110257613
110258586	2.356	0.614	-1.9616428	1.24E-21	4.35E-22	LOC110258586
110258588	0.844	0.234	-1.7268071	1.87E-08	1.28E-08	LOC110258588
110258627	0.252	0.066	-1.9657206	0.00073687	0.000813671	LOC110258627
110258836	0.114	0.02	-2.38304	0.000844565	0.000939932	LOC110258836
110258908	0.116	0.03	-1.7881144	9.29E-05	9.10E-05	LOC110258908
110258978	0.418	0.114	-1.8483285	4.84E-05	4.58E-05	LOC110258978
110259135	7.618	3.828	-1.0347659	5.19E-28	1.51E-28	LOC110259135
110259856	2.014	0.326	-2.642071	2.74E-49	5.01E-50	LOC110259856
110259937	0.84	0.344	-1.1958493	0.000840858	0.000935563	LOC110259937
110260296	0.698	0.314	-1.0668825	0.000665916	0.000731173	ALKAL1
110260309	1.232	0.54	-1.2648107	8.96E-07	7.09E-07	LOC110260309
110260351	1.814	0.894	-1.0110508	0.000485112	0.000521336	LOC110260351
110260907	1.702	0.608	-1.4862216	2.07E-62	3.11E-63	LOC110260907
110261137	0.998	0.342	-1.4997783	1.48E-09	9.35E-10	GJB3
110261159	0.858	0.398	-1.0588862	8.55E-11	4.97E-11	PRELID3A
110261274	0.104	0	-4.8642477	8.18E-05	7.96E-05	WTIP
110261361	0.21	0.022	-3.1272821	4.45E-09	2.91E-09	LOC110261361
110261410	0.99	0.432	-1.2329916	3.99E-06	3.37E-06	LOC110261410
110261553	4.7	0.782	-2.5827566	1.27E-30	3.44E-31	LOC110261553
110262053	9.862	3.124	-1.6115224	8.52E-122	7.30E-123	LOC110262053
110262192	1.64	0	-6.5365976	2.64E-12	1.40E-12	LOC110262192
110262277	0.196	0.04	-2.3509415	5.12E-05	4.87E-05	UBTFL1
396579	0.214	0	-4.5423196	0.000460596	0.000492921	TNNT1
396643	0.112	0.076	-1.5250416	0.000180086	0.000182106	SSTR3
396711	5.26	1.876	-1.4804576	9.13E-136	7.00E-137	LOC396711
396725	0.372	0.15	-1.4850948	5.96E-05	5.71E-05	DES
396729	0.348	0.124	-1.5285138	8.83E-09	5.91E-09	TEK
396735	183.656	71.62	-1.4120486	0	0	CXCL16
396811	1.44	0.714	-1.0531132	5.08E-13	2.59E-13	PTGER3
396845	2.502	1.112	-1.2217288	3.96E-20	1.47E-20	TF
396847	0.162	0.056	-1.4820327	0.000607328	0.000662532	MAN2B2

Gene ID	F1 FPKM	F3 FPKM	log2(F3/F1)	Qvalue(F1-vs-F3)	Pvalue(F1-vs-F3)	Other Gene ID
396848	3.078	1.978	-1.1059283	3.00E-47	5.67E-48	MYLK
396856	0.046	0.02	-1.2792852	0.000743923	0.000822214	RYR2
396859	15.642	2.908	-2.4412473	0	0	NOS2
396888	0.164	0.066	-1.2843384	3.41E-09	2.21E-09	RYR3
396896	1.138	0.274	-2.0094456	5.67E-16	2.49E-16	CHAT
396940	0.086	0.004	-4.2016059	0.000652668	0.000715871	AMELX
396954	0.72	0.336	-1.06925	0.000381098	0.000402985	PROC
396957	3.144	0.752	-2.2114951	2.68E-35	6.42E-36	DCN
396963	0.778	0.292	-1.4204442	6.10E-09	4.03E-09	ITIH1
396971	0.51	0.156	-1.6896704	7.46E-10	4.61E-10	ALOX15
397048	0.388	0.062	-2.6577968	6.08E-09	4.02E-09	SRD5A2
397073	88.674	41.252	-1.1072257	0	0	ABCG2
397095	33.154	15.492	-1.0839241	2.05E-174	1.23E-175	GGT1
397113	405.52	184.368	-1.1489381	0	0	SLC5A1
397124	2.284	1.152	-1.0574346	1.70E-07	1.26E-07	RBP4
397152	9.288	3.976	-1.2325183	1.48E-115	1.32E-116	TMPRSS15
397160	0.278	0.144	-1.0948607	0.000966882	0.001083484	CD34
397172	0.232	0.14	-1.1429818	3.86E-05	3.61E-05	CALCRL
397192	0.83	0.164	-2.3358688	3.77E-11	2.14E-11	VTN
397200	29.232	13.906	-1.0775687	1.78E-216	9.08E-218	SLC28A1
397206	3.214	0.992	-1.7027843	7.82E-11	4.53E-11	MGP
397216	0.102	0.022	-2.1797495	0.000120832	0.000119807	OPRD1
397246	23.054	9.73	-1.2484921	5.15E-124	4.31E-125	VNN1
397309	11.696	5.25	-1.2677895	2.17E-113	1.98E-114	HTR1D
397311	0.394	0.202	-1.0680012	1.82E-07	1.35E-07	KDR
397322	1.23	0.594	-1.0162508	4.92E-07	3.81E-07	IGFALS
397346	5.17	1.624	-1.6625859	7.62E-73	1.02E-73	MMP13
397358	50.694	25.194	-1.0147776	0	0	SLC9A3
397376	31.406	13.522	-1.2325247	9.38E-203	5.17E-204	SLC5A4
397381	702.696	334.056	-1.0676292	0	0	MTTP
397419	4.09	1.802	-1.1165557	2.89E-07	2.19E-07	TTR
397460	0.252	0.116	-1.2088959	2.24E-06	1.84E-06	TNC
397474	6.578	1.588	-2.0090192	1.64E-83	1.96E-84	F12
397526	0.77	0.296	-1.519236	4.13E-10	2.52E-10	C7

Gene ID	F1 FPKM	F3 FPKM	log2(F3/F1)	Qvalue(F1-vs-F3)	Pvalue(F1-vs-F3)	Other Gene ID
397535	32.514	14.888	-1.1300278	0	0	ABCC2
397541	3.218	0.694	-2.1301937	1.54E-56	2.51E-57	PTGS1
397583	12.594	5.254	-1.2524776	1.09E-123	9.13E-125	LIPE
397621	0.544	0.278	-1.0513668	2.34E-05	2.13E-05	SLC17A1
397624	79.636	24.82	-1.6911512	0	0	SLC15A1
397626	0.616	0.274	-1.3979297	2.60E-07	1.96E-07	LYVE1
397628	8.976	4.064	-1.124747	4.34E-45	8.50E-46	ANGPTL4
397681	2517.77	828.416	-1.6137408	0	0	APOA4
397691	6715.18	2696.27	-1.3195893	0	0	APOA1
399528	4.606	1.15	-2.0044057	2.48E-78	3.18E-79	CPT1B
399543	0.418	0.216	-1.0910356	6.86E-11	3.96E-11	VWF
403104	14.086	5.938	-1.2777319	5.94E-128	4.84E-129	CYP2B22
403106	17.064	6.09	-1.5091093	7.42E-144	5.31E-145	CYP2C32
403108	129.006	44.644	-1.5327563	0	0	CYP2C34
403110	12.82	4.784	-1.4334977	5.60E-97	5.83E-98	CYP2C36
403215	35.194	15.24	-1.2223922	2.42E-215	1.24E-216	CYP2C49
403324	256.198	69.496	-1.8768519	0	0	CYP3A29
404690	0.566	0.266	-1.082352	0.000944749	0.001057588	ST6GALNAC5
404693	0.746	0.302	-1.4014571	1.07E-08	7.24E-09	CAV1
404698	91.34	44.964	-1.012634	0	0	HYAL1
406187	3342.59	1243.49	-1.4515452	0	0	APOC3
407066	51.798	22.798	-1.2602043	1.29E-75	1.69E-76	HBB
414411	106.336	36.64	-1.5546645	0	0	ASS1
414737	0.394	0.11	-1.9723075	3.32E-11	1.88E-11	CDH5
414836	0.246	0.088	-1.5324718	8.98E-12	4.91E-12	FBN1
414914	17.676	6.376	-1.4696529	0	0	COL17A1
445003	4.302	1.618	-1.40011	3.39E-89	3.78E-90	MRC1
445453	0.586	0	-4.764712	0.000145416	0.000145404	CCL27
445536	2.164	1.134	-1.0400148	0.000272704	0.000282993	LOC445536
492312	0.362	0.128	-1.635429	0.000637087	0.000697857	MADCAM1
494019	71.496	31.832	-1.1609148	2.42E-251	1.07E-252	CXCL10
494460	0.386	0.106	-2.0353596	1.97E-05	1.78E-05	CXCL12
494464	1.16	0.196	-2.6943227	0.000231101	0.000237343	CCL19
497235	1.76	0.572	-1.6205855	3.01E-18	1.21E-18	SLC5A10

Gene ID	F1 FPKM	F3 FPKM	log2(F3/F1)	Qvalue(F1-vs-F3)	Pvalue(F1-vs-F3)	Other Gene ID
595124	5.596	2.676	-1.1580882	8.18E-31	2.20E-31	SPARC
613133	206.692	98.302	-1.1767031	0	0	ACE
733615	2.434	1.2	-1.1131979	1.68E-09	1.06E-09	ACTA2
733628	0.212	0.074	-1.5423196	2.65E-05	2.43E-05	AEBP1
733634	3.418	1.542	-1.1450629	9.25E-15	4.30E-15	AKR1C1
733662	4.478	0.918	-2.2351819	1.26E-54	2.11E-55	F10
733694	1.974	0.968	-1.0058678	4.97E-14	2.40E-14	AKR1C1
