# Supplementary material for: Transcriptome Profile Analysis of Intestinal Upper Villus Epithelial Cells and Crypt Epithelial Cells of Suckling Piglets
Source: Animals (Basel). 2022 Sep 7;12(18):2324. doi: 10.3390/ani12182324 (PMC9494997; doi:10.3390/ani12182324)
Supplement: Supplementary file 1 [file animals-12-02324-s001.zip › Table S3.KEGG Pathway classification of DEGs.pdf]

Table S3. KEGG Pathway classification of DEGs.

Pathway ID	Pathway Name	Level 1	Level 2	Term Candidate Gene Num	Total Candidate Gene Num	Term Gene Num	Total Gene Num	Rich Ratio	P value	Q value
ko04060	Cytokine-cytokine receptor interaction	Environmental Information Processing	Signaling molecules and interaction	44	441	325	11608	0.1353846	1.40E-13	3.99E-11
ko00830	Retinol metabolism	Metabolism	Metabolism of cofactors and	16	441	86	11608	0.1860465	1.20E-07	1.71E-05
ko05204	Chemical carcinogenesis	Human Diseases	Cancers: Overview	15	441	86	11608	0.1744186	7.15E-07	6.80E-05
ko04975	Fat digestion and absorption	Organismal Systems	Digestive system	11	441	53	11608	0.2075472	3.78E-06	0.00026946
ko00140	Steroid hormone biosynthesis	Metabolism	Lipid metabolism	12	441	72	11608	0.1666667	1.51E-05	0.00085929
ko00590	Arachidonic acid metabolism	Metabolism	Lipid metabolism	14	441	101	11608	0.1386139	2.65E-05	0.00094275
ko05143	African trypanosomiasis	Human Diseases	Infectious diseases: Parasitic	10	441	53	11608	0.1886792	2.54E-05	0.00094275
ko05323	Rheumatoid arthritis	Human Diseases	Immune diseases	16	441	125	11608	0.128	1.99E-05	0.00094275
ko00591	Linoleic acid metabolism	Metabolism	Lipid metabolism	9	441	44	11608	0.2045455	3.29E-05	0.00104325
ko04672	Intestinal immune network for IgA	Organismal Systems	Immune system	11	441	68	11608	0.1617647	4.55E-05	0.00108098
ko05144	Malaria	Human Diseases	Infectious diseases: Parasitic	12	441	80	11608	0.15	4.49E-05	0.00108098
ko05340	Primary immunodeficiency	Human Diseases	Immune diseases	10	441	56	11608	0.1785714	4.19E-05	0.00108098
ko04062	Chemokine signaling pathway	Organismal Systems	Immune system	22	441	254	11608	0.0866142	0.0002721	0.00553888
ko04726	Serotonergic synapse	Organismal Systems	Nervous system	15	441	139	11608	0.1079137	0.0002544	0.00553888
ko05321	Inflammatory bowel disease (IBD)	Human Diseases	Immune diseases	11	441	87	11608	0.1264368	0.0004351	0.0082664
ko04064	NF-kappa B signaling pathway	Environmental Information Processing	Signal transduction	14	441	139	11608	0.1007194	0.000814	0.01449997

Pathway ID	Pathway Name	Level 1	Level 2	Term Candidate Gene Num	Total Candidate Gene Num	Term Gene Num	Total Gene Num	Rich Ratio	P value	Q value
ko05140	Leishmaniasis	Human Diseases	Infectious diseases: Parasitic	11	441	95	11608	0.1157895	0.0009245	0.01549892
ko03320	PPAR signaling pathway	Organismal Systems	Endocrine system	11	441	96	11608	0.1145833	0.0010093	0.01598096
ko04610	Complement and coagulation cascades	Organismal Systems	Immune system	13	441	131	11608	0.0992366	0.0014181	0.02127093
ko04979	Cholesterol metabolism	Organismal Systems	Digestive system	9	441	75	11608	0.12	0.0020643	0.02941559
ko04640	Hematopoietic cell lineage	Organismal Systems	Immune system	12	441	122	11608	0.0983607	0.0023031	0.03125647
ko04657	IL-17 signaling pathway	Organismal Systems	Immune system	18	441	227	11608	0.0792952	0.0025806	0.03343113
ko04614	Renin-angiotensin system	Organismal Systems	Endocrine system	5	441	28	11608	0.1785714	0.0036877	0.04569538
ko04668	TNF signaling pathway	Environmental Information Processing	Signal transduction	12	441	137	11608	0.0875912	0.005931	0.07043038
ko04512	ECM-receptor interaction	Environmental Information Processing	Signaling molecules and interaction	22	441	326	11608	0.0674847	0.0065855	0.07507504
ko04151	PI3K-Akt signaling pathway	Environmental Information Processing	Signal transduction	37	441	641	11608	0.0577223	0.0071953	0.07887176
ko04625	C-type lectin receptor signaling pathway	Organismal Systems	Immune system	12	441	142	11608	0.084507	0.0078405	0.08276088
ko04350	TGF-beta signaling pathway	Environmental Information Processing	Signal transduction	9	441	97	11608	0.0927835	0.011329	0.115313
ko04371	Apelin signaling pathway	Environmental Information Processing	Signal transduction	14	441	188	11608	0.0744681	0.0125114	0.1229568
ko05322	Systemic lupus erythematosus	Human Diseases	Immune diseases	12	441	152	11608	0.0789474	0.0130799	0.1242593
ko04659	Th17 cell differentiation	Organismal Systems	Immune system	11	441	136	11608	0.0808824	0.0144895	0.1332096
ko04977	Vitamin digestion and absorption	Organismal Systems	Digestive system	5	441	39	11608	0.1282051	0.0153677	0.1368681
ko04928	Parathyroid hormone synthesis, secretion and	Organismal Systems	Endocrine system	13	441	178	11608	0.0730337	0.0182473	0.1575906

Pathway ID	Pathway Name	Level 1	Level 2	Term Candidate Gene Num	Total Candidate Gene Num	Term Gene Num	Total Gene Num	Rich Ratio	P value	Q value
ko04010	MAPK signaling pathway	Environmental Information Processing	Signal transduction	22	441	361	11608	0.0609418	0.0197802	0.1610671
ko04974	Protein digestion and absorption	Organismal Systems	Digestive system	37	441	686	11608	0.0539359	0.0194964	0.1610671
ko04923	Regulation of lipolysis in adipocytes	Organismal Systems	Endocrine system	7	441	74	11608	0.0945946	0.0219818	0.1740224
ko05146	Amoebiasis	Human Diseases	Infectious diseases: Parasitic	30	441	547	11608	0.0548446	0.0275226	0.2119983
ko04068	FoxO signaling pathway	Environmental Information Processing	Signal transduction	11	441	153	11608	0.0718954	0.0315034	0.2244617
ko04510	Focal adhesion	Cellular Processes	Cellular community - eukaryotes	27	441	487	11608	0.0554415	0.0314629	0.2244617
ko05202	Transcriptional misregulation in cancer	Human Diseases	Cancers: Overview	17	441	273	11608	0.0622711	0.0314661	0.2244617
ko04360	Axon guidance	Organismal Systems	Development	16	441	255	11608	0.0627451	0.034089	0.2278475
ko04913	Ovarian steroidogenesis	Organismal Systems	Endocrine system	6	441	64	11608	0.09375	0.0341029	0.2278475
ko05142	Chagas disease (American	Human Diseases	Infectious diseases: Parasitic	10	441	136	11608	0.0735294	0.034377	0.2278475
ko05320	Autoimmune thyroid disease	Human Diseases	Immune diseases	8	441	100	11608	0.08	0.0362755	0.2349662
ko00430	Taurine and hypotaurine metabolism	Metabolism	Metabolism of other amino acids	3	441	20	11608	0.15	0.0384089	0.2411847
ko05132	Salmonella infection	Human Diseases	Infectious diseases: Bacterial	12	441	178	11608	0.0674157	0.0389281	0.2411847
ko05200	Pathways in cancer	Human Diseases	Cancers: Overview	37	441	726	11608	0.0509642	0.0411471	0.2495089
ko04620	Toll-like receptor signaling pathway	Organismal Systems	Immune system	10	441	144	11608	0.0694444	0.0477332	0.2720791
ko04742	Taste transduction	Organismal Systems	Sensory system	7	441	87	11608	0.0804598	0.0472071	0.2720791
ko05414	Dilated cardiomyopathy (DCM)	Human Diseases	Cardiovascular diseases	10	441	144	11608	0.0694444	0.0477332	0.2720791

Pathway ID	Pathway Name	Level 1	Level 2	Term Candidate Gene Num	Total Candidate Gene Num	Term Gene Num	Total Gene Num	Rich Ratio	P value	Q value
ko05133	Pertussis	Human Diseases	Infectious diseases: Bacterial	8	441	107	11608	0.0747664	0.0507421	0.2781055
ko05410	Hypertrophic cardiomyopathy (HCM)	Human Diseases	Cardiovascular diseases	9	441	126	11608	0.0714286	0.0505662	0.2781055
ko04380	Osteoclast differentiation	Organismal Systems	Development	11	441	167	11608	0.0658683	0.0536099	0.2829413
ko05210	Colorectal cancer	Human Diseases	Cancers: Specific types	8	441	108	11608	0.0740741	0.0530735	0.2829413
ko04978	Mineral absorption	Organismal Systems	Digestive system	5	441	55	11608	0.0909091	0.057007	0.2954001
ko04660	T cell receptor signaling pathway	Organismal Systems	Immune system	9	441	130	11608	0.0692308	0.0593968	0.302287
ko00500	Starch and sucrose metabolism	Metabolism	Carbohydrate metabolism	4	441	40	11608	0.1	0.0641109	0.3174274
ko04216	Ferroptosis	Cellular Processes	Cell growth and death	5	441	57	11608	0.0877193	0.0645993	0.3174274
ko05020	Prion diseases	Human Diseases	Neurodegenerative diseases	5	441	58	11608	0.0862069	0.0685978	0.3285178
ko05145	Toxoplasmosis	Human Diseases	Infectious diseases: Parasitic	9	441	134	11608	0.0671642	0.0691617	0.3285178
ko04390	Hippo signaling pathway	Environmental Information Processing	Signal transduction	12	441	197	11608	0.0609137	0.0726522	0.3394406
ko04662	B cell receptor signaling pathway	Organismal Systems	Immune system	7	441	97	11608	0.0721649	0.0757419	0.3426418
ko05418	Fluid shear stress and atherosclerosis	Human Diseases	Cardiovascular diseases	12	441	198	11608	0.0606061	0.074827	0.3426418
ko04926	Relaxin signaling pathway	Organismal Systems	Endocrine system	21	441	396	11608	0.0530303	0.0771949	0.3437585
ko02010	ABC transporters	Environmental Information Processing	Membrane transport	6	441	81	11608	0.0740741	0.0872257	0.3824513
ko05134	Legionellosis	Human Diseases	Infectious diseases: Bacterial	7	441	103	11608	0.0679612	0.0968366	0.4181579
ko00480	Glutathione metabolism	Metabolism	Metabolism of other amino acids	5	441	65	11608	0.0769231	0.1002799	0.4265638

Pathway ID	Pathway Name	Level 1	Level 2	Term Candidate Gene Num	Total Candidate Gene Num	Term Gene Num	Total Gene Num	Rich Ratio	P value	Q value
ko04950	Maturity onset diabetes of the young	Human Diseases	Endocrine and metabolic diseases	3	441	30	11608	0.1	0.1039461	0.4350046
ko05330	Allograft rejection	Human Diseases	Immune diseases	5	441	66	11608	0.0757576	0.1053169	0.4350046
ko04514	Cell adhesion molecules (CAMs)	Environmental Information Processing	Signaling molecules and interaction	11	441	191	11608	0.0575916	0.1117053	0.4504148
ko05167	Kaposi's sarcoma-associated herpesvirus	Human Diseases	Infectious diseases: Viral	14	441	257	11608	0.0544747	0.1122086	0.4504148
ko00760	Nicotinate and nicotinamide metabolism	Metabolism	Metabolism of cofactors and	4	441	49	11608	0.0816327	0.1146499	0.4538225
ko05211	Renal cell carcinoma	Human Diseases	Cancers: Specific types	6	441	88	11608	0.0681818	0.1176097	0.4591612
ko04973	Carbohydrate digestion and absorption	Organismal Systems	Digestive system	4	441	51	11608	0.0784314	0.1277158	0.48532
ko05310	Asthma	Human Diseases	Immune diseases	4	441	51	11608	0.0784314	0.1277158	0.48532
ko04713	Circadian entrainment	Organismal Systems	Environmental adaptation	8	441	133	11608	0.0601504	0.1336731	0.4884209
ko04933	AGE-RAGE signaling pathway in diabetic	Human Diseases	Endocrine and metabolic diseases	18	441	355	11608	0.0507042	0.130502	0.4884209
ko05222	Small cell lung cancer	Human Diseases	Cancers: Specific types	10	441	176	11608	0.0568182	0.1333889	0.4884209
ko04976	Bile secretion	Organismal Systems	Digestive system	7	441	115	11608	0.0608696	0.1476146	0.5325337
ko05206	MicroRNAs in cancer	Human Diseases	Cancers: Overview	11	441	203	11608	0.0541872	0.150623	0.5365944
ko05120	Epithelial cell signaling in Helicobacter pylori	Human Diseases	Infectious diseases: Bacterial	5	441	75	11608	0.0666667	0.1558782	0.5484603
ko04921	Oxytocin signaling pathway	Organismal Systems	Endocrine system	11	441	206	11608	0.0533981	0.1613281	0.5607135
ko04630	Jak-STAT signaling pathway	Environmental Information Processing	Signal transduction	11	441	208	11608	0.0528846	0.1686711	0.5791718
ko00561	Glycerolipid metabolism	Metabolism	Lipid metabolism	5	441	79	11608	0.0632911	0.1810499	0.6070497

Pathway ID	Pathway Name	Level 1	Level 2	Term Candidate Gene Num	Total Candidate Gene Num	Term Gene Num	Total Gene Num	Rich Ratio	P value	Q value
ko05220	Chronic myeloid leukemia	Human Diseases	Cancers: Specific types	6	441	100	11608	0.06	0.1800306	0.6070497
ko05152	Tuberculosis	Human Diseases	Infectious diseases: Bacterial	14	441	282	11608	0.0496454	0.1864576	0.6179118
ko00220	Arginine biosynthesis	Metabolism	Amino acid metabolism	2	441	21	11608	0.0952381	0.1888648	0.618695
ko00790	Folate biosynthesis	Metabolism	Metabolism of cofactors and	3	441	40	11608	0.075	0.1934572	0.6265375
ko05205	Proteoglycans in cancer	Human Diseases	Cancers: Overview	13	441	262	11608	0.0496183	0.1980511	0.6271618
ko00770	Pantothenate and CoA biosynthesis	Metabolism	Metabolism of cofactors and	2	441	22	11608	0.0909091	0.2028424	0.630885
ko04024	cAMP signaling pathway	Environmental Information Processing	Signal transduction	14	441	287	11608	0.0487805	0.2036541	0.630885
ko05030	Cocaine addiction	Human Diseases	Substance dependence	4	441	62	11608	0.0645161	0.2090004	0.6404851
ko03020	RNA polymerase	Genetic Information Processing	Transcription	6	441	107	11608	0.0560748	0.2214677	0.6584302
ko04270	Vascular smooth muscle contraction	Organismal Systems	Circulatory system	9	441	175	11608	0.0514286	0.221787	0.6584302
ko04621	NOD-like receptor signaling pathway	Organismal Systems	Immune system	12	441	244	11608	0.0491803	0.2184957	0.6584302
ko05166	HTLV-I infection	Human Diseases	Infectious diseases: Viral	15	441	318	11608	0.0471698	0.2293862	0.6739698
ko04215	Apoptosis - multiple species	Cellular Processes	Cell growth and death	3	441	44	11608	0.0681818	0.2333894	0.6787345
ko00260	Glycine, serine and threonine metabolism	Metabolism	Amino acid metabolism	3	441	45	11608	0.0666667	0.2436001	0.6942603
ko00860	Porphyrin and chlorophyll metabolism	Metabolism	Metabolism of cofactors and	3	441	45	11608	0.0666667	0.2436001	0.6942603
ko01523	Antifolate resistance	Human Diseases	Drug resistance: Antineoplastic	4	441	67	11608	0.0597015	0.2499103	0.7051924
ko00360	Phenylalanine metabolism	Metabolism	Amino acid metabolism	2	441	26	11608	0.0769231	0.2595852	0.7253116

Pathway ID	Pathway Name	Level 1	Level 2	Term Candidate Gene Num	Total Candidate Gene Num	Term Gene Num	Total Gene Num	Rich Ratio	P value	Q value
ko04920	Adipocytokine signaling pathway	Organismal Systems	Endocrine system	5	441	91	11608	0.0549451	0.2640417	0.7306008
ko04310	Wnt signaling pathway	Environmental Information Processing	Signal transduction	9	441	185	11608	0.0486486	0.2710244	0.7427111
ko05219	Bladder cancer	Human Diseases	Cancers: Specific types	3	441	48	11608	0.0625	0.2746135	0.7453795
ko05169	Epstein-Barr virus infection	Human Diseases	Infectious diseases: Viral	14	441	307	11608	0.0456026	0.278862	0.7497705
ko00400	Phenylalanine, tyrosine and tryptophan	Metabolism	Amino acid metabolism	1	441	9	11608	0.1111111	0.2943996	0.7841485
ko04964	Proximal tubule bicarbonate reclamation	Organismal Systems	Excretory system	2	441	29	11608	0.0689655	0.3023241	0.7899574
ko05033	Nicotine addiction	Human Diseases	Substance dependence	3	441	51	11608	0.0588235	0.3059973	0.7899574
ko05100	Bacterial invasion of epithelial cells	Human Diseases	Infectious diseases: Bacterial	8	441	168	11608	0.047619	0.3076676	0.7899574
ko05217	Basal cell carcinoma	Human Diseases	Cancers: Specific types	4	441	73	11608	0.0547945	0.3009346	0.7899574
ko05224	Breast cancer	Human Diseases	Cancers: Specific types	9	441	193	11608	0.0466321	0.3125335	0.7952861
ko05150	Staphylococcus aureus infection	Human Diseases	Infectious diseases: Bacterial	4	441	75	11608	0.0533333	0.318228	0.8026104
ko00740	Riboflavin metabolism	Metabolism	Metabolism of cofactors and	1	441	10	11608	0.1	0.3212269	0.8030673
ko00040	Pentose and glucuronate interconversions	Metabolism	Carbohydrate metabolism	2	441	31	11608	0.0645161	0.3305966	0.8037422
ko04623	Cytosolic DNA-sensing pathway	Organismal Systems	Immune system	5	441	100	11608	0.05	0.330987	0.8037422
ko05162	Measles	Human Diseases	Infectious diseases: Viral	9	441	197	11608	0.0456853	0.3337997	0.8037422
ko05213	Endometrial cancer	Human Diseases	Cancers: Specific types	4	441	77	11608	0.0519481	0.3355976	0.8037422
ko05412	Arrhythmogenic right ventricular	Human Diseases	Cardiovascular diseases	5	441	100	11608	0.05	0.330987	0.8037422

Pathway ID	Pathway Name	Level 1	Level 2	Term Candidate Gene Num	Total Candidate Gene Num	Term Gene Num	Total Gene Num	Rich Ratio	P value	Q value
ko00650	Butanoate metabolism	Metabolism	Carbohydrate metabolism	2	441	33	11608	0.0606061	0.3585404	0.8463374
ko04725	Cholinergic synapse	Organismal Systems	Nervous system	6	441	128	11608	0.046875	0.3593222	0.8463374
ko00230	Purine metabolism	Metabolism	Nucleotide metabolism	12	441	279	11608	0.0430108	0.3714439	0.8677173
ko04924	Renin secretion	Organismal Systems	Endocrine system	5	441	106	11608	0.0471698	0.3765154	0.8724137
ko00601	Glycosphingolipid biosynthesis - lacto and	Metabolism	Glycan biosynthesis and metabolism	2	441	37	11608	0.0540541	0.4130276	0.9300616
ko04650	Natural killer cell mediated cytotoxicity	Organismal Systems	Immune system	7	441	161	11608	0.0434783	0.4128457	0.9300616
ko05032	Morphine addiction	Human Diseases	Substance dependence	5	441	111	11608	0.045045	0.4144485	0.9300616
ko05416	Viral myocarditis	Human Diseases	Cardiovascular diseases	5	441	111	11608	0.045045	0.4144485	0.9300616
ko04972	Pancreatic secretion	Organismal Systems	Digestive system	6	441	137	11608	0.0437956	0.4207749	0.9345437
ko05161	Hepatitis B	Human Diseases	Infectious diseases: Viral	8	441	188	11608	0.0425532	0.423004	0.9345437
ko00072	Synthesis and degradation of ketone	Metabolism	Lipid metabolism	1	441	15	11608	0.0666667	0.4408459	0.93762
ko04066	HIF-1 signaling pathway	Environmental Information Processing	Signal transduction	5	441	114	11608	0.0438596	0.437052	0.93762
ko04122	Sulfur relay system	Genetic Information Processing	Folding, sorting and degradation	1	441	15	11608	0.0666667	0.4408459	0.93762
ko05212	Pancreatic cancer	Human Diseases	Cancers: Specific types	4	441	88	11608	0.0454545	0.4308994	0.93762
ko05226	Gastric cancer	Human Diseases	Cancers: Specific types	15	441	370	11608	0.0405405	0.4356996	0.93762
ko04724	Glutamatergic synapse	Organismal Systems	Nervous system	6	441	141	11608	0.0425532	0.4479116	0.9455912
ko00061	Fatty acid biosynthesis	Metabolism	Lipid metabolism	1	441	16	11608	0.0625	0.4621163	0.9468596

Pathway ID	Pathway Name	Level 1	Level 2	Term Candidate Gene Num	Total Candidate Gene Num	Term Gene Num	Total Gene Num	Rich Ratio	P value	Q value
ko00250	Alanine, aspartate and glutamate metabolism	Metabolism	Amino acid metabolism	2	441	41	11608	0.0487805	0.465124	0.9468596
ko04020	Calcium signaling pathway	Environmental Information Processing	Signal transduction	11	441	272	11608	0.0404412	0.4598547	0.9468596
ko04115	p53 signaling pathway	Cellular Processes	Cell growth and death	5	441	117	11608	0.042735	0.4594565	0.9468596
ko05163	Human cytomegalovirus infection	Human Diseases	Infectious diseases: Viral	13	441	325	11608	0.04	0.4649296	0.9468596
ko00052	Galactose metabolism	Metabolism	Carbohydrate metabolism	2	441	42	11608	0.047619	0.4777193	0.9588028
ko04392	Hippo signaling pathway - multiple species	Environmental Information Processing	Signal transduction	2	441	42	11608	0.047619	0.4777193	0.9588028
ko00730	Thiamine metabolism	Metabolism	Metabolism of cofactors and	1	441	18	11608	0.0555556	0.5022654	0.994742
ko05031	Amphetamine addiction	Human Diseases	Substance dependence	4	441	97	11608	0.0412371	0.5060968	0.994742
ko05165	Human papillomavirus infection	Human Diseases	Infectious diseases: Viral	25	441	650	11608	0.0384615	0.5047784	0.994742
ko00010	Glycolysis / Gluconeogenesis	Metabolism	Carbohydrate metabolism	3	441	102	11608	0.0294118	0.7499366	1
ko00020	Citrate cycle (TCA cycle)	Metabolism	Carbohydrate metabolism	1	441	51	11608	0.0196078	0.8618835	1
ko00051	Fructose and mannose metabolism	Metabolism	Carbohydrate metabolism	1	441	36	11608	0.0277778	0.7525341	1
ko00053	Ascorbate and aldarate metabolism	Metabolism	Carbohydrate metabolism	1	441	26	11608	0.0384615	0.6350981	1
ko00071	Fatty acid degradation	Metabolism	Lipid metabolism	2	441	57	11608	0.0350877	0.6432413	1
ko00100	Steroid biosynthesis	Metabolism	Lipid metabolism	1	441	29	11608	0.0344828	0.6752168	1
ko00120	Primary bile acid biosynthesis	Metabolism	Lipid metabolism	1	441	20	11608	0.05	0.539424	1
ko00240	Pyrimidine metabolism	Metabolism	Nucleotide metabolism	7	441	189	11608	0.037037	0.581503	1

Pathway ID	Pathway Name	Level 1	Level 2	Term Candidate Gene Num	Total Candidate Gene Num	Term Gene Num	Total Gene Num	Rich Ratio	P value	Q value
ko00270	Cysteine and methionine metabolism	Metabolism	Amino acid metabolism	2	441	57	11608	0.0350877	0.6432413	1
ko00280	Valine, leucine and isoleucine degradation	Metabolism	Amino acid metabolism	2	441	70	11608	0.0285714	0.7507536	1
ko00310	Lysine degradation	Metabolism	Amino acid metabolism	2	441	132	11608	0.0151515	0.9633254	1
ko00330	Arginine and proline metabolism	Metabolism	Amino acid metabolism	2	441	70	11608	0.0285714	0.7507536	1
ko00350	Tyrosine metabolism	Metabolism	Amino acid metabolism	2	441	53	11608	0.0377358	0.6035308	1
ko00380	Tryptophan metabolism	Metabolism	Amino acid metabolism	2	441	59	11608	0.0338983	0.6618902	1
ko00410	beta-Alanine metabolism	Metabolism	Metabolism of other amino acids	1	441	37	11608	0.027027	0.7619648	1
ko00511	Other glycan degradation	Metabolism	Glycan biosynthesis and metabolism	1	441	26	11608	0.0384615	0.6350981	1
ko00512	Mucin type O-glycan biosynthesis	Metabolism	Glycan biosynthesis and metabolism	1	441	35	11608	0.0285714	0.7427306	1
ko00531	Glycosaminoglycan degradation	Metabolism	Glycan biosynthesis and metabolism	1	441	24	11608	0.0416667	0.6056422	1
ko00532	Glycosaminoglycan biosynthesis - chondroitin	Metabolism	Glycan biosynthesis and metabolism	1	441	23	11608	0.0434783	0.5900363	1
ko00564	Glycerophospholipid metabolism	Metabolism	Lipid metabolism	4	441	135	11608	0.0296296	0.7597137	1
ko00565	Ether lipid metabolism	Metabolism	Lipid metabolism	2	441	65	11608	0.0307692	0.7131525	1
ko00592	alpha-Linolenic acid metabolism	Metabolism	Lipid metabolism	1	441	32	11608	0.03125	0.7109337	1
ko00600	Sphingolipid metabolism	Metabolism	Lipid metabolism	2	441	64	11608	0.03125	0.7050855	1
ko00603	Glycosphingolipid biosynthesis - globo and	Metabolism	Glycan biosynthesis and metabolism	1	441	19	11608	0.0526316	0.5212042	1
ko00604	Glycosphingolipid biosynthesis - ganglio	Metabolism	Glycan biosynthesis and metabolism	1	441	22	11608	0.0454545	0.5738143	1

Pathway ID	Pathway Name	Level 1	Level 2	Term Candidate Gene Num	Total Candidate Gene Num	Term Gene Num	Total Gene Num	Rich Ratio	P value	Q value
ko00620	Pyruvate metabolism	Metabolism	Carbohydrate metabolism	3	441	88	11608	0.0340909	0.655311	1
ko00640	Propanoate metabolism	Metabolism	Carbohydrate metabolism	1	441	66	11608	0.0151515	0.9229735	1
ko00980	Metabolism of xenobiotics by	Metabolism	Xenobiotics biodegradation and	2	441	66	11608	0.030303	0.7210339	1
ko00982	Drug metabolism - cytochrome P450	Metabolism	Xenobiotics biodegradation and	2	441	63	11608	0.031746	0.6968307	1
ko00983	Drug metabolism - other enzymes	Metabolism	Xenobiotics biodegradation and	2	441	78	11608	0.025641	0.8020848	1
ko01212	Fatty acid metabolism	Metabolism	Global and overview maps	2	441	60	11608	0.0333333	0.6709174	1
ko01230	Biosynthesis of amino acids	Metabolism	Global and overview maps	1	441	90	11608	0.0111111	0.9697885	1
ko01521	EGFR tyrosine kinase inhibitor resistance	Human Diseases	Drug resistance: Antineoplastic	2	441	99	11608	0.020202	0.8948947	1
ko01522	Endocrine resistance	Human Diseases	Drug resistance: Antineoplastic	5	441	144	11608	0.0347222	0.6438103	1
ko01524	Platinum drug resistance	Human Diseases	Drug resistance: Antineoplastic	3	441	91	11608	0.032967	0.6775436	1
ko03008	Ribosome biogenesis in eukaryotes	Genetic Information Processing	Translation	1	441	106	11608	0.009434	0.9838299	1
ko03010	Ribosome	Genetic Information Processing	Translation	2	441	191	11608	0.0104712	0.9950078	1
ko03013	RNA transport	Genetic Information Processing	Translation	6	441	279	11608	0.0215054	0.9572116	1
ko03015	mRNA surveillance pathway	Genetic Information Processing	Translation	7	441	189	11608	0.037037	0.581503	1
ko03018	RNA degradation	Genetic Information Processing	Folding, sorting and degradation	2	441	101	11608	0.019802	0.9012147	1
ko03022	Basal transcription factors	Genetic Information Processing	Transcription	2	441	147	11608	0.0136054	0.9776611	1
ko03040	Spliceosome	Genetic Information Processing	Transcription	1	441	188	11608	0.0053191	0.9993522	1

Pathway ID	Pathway Name	Level 1	Level 2	Term Candidate Gene Num	Total Candidate Gene Num	Term Gene Num	Total Gene Num	Rich Ratio	P value	Q value
ko03410	Base excision repair	Genetic Information Processing	Replication and repair	1	441	48	11608	0.0208333	0.8447876	1
ko03440	Homologous recombination	Genetic Information Processing	Replication and repair	1	441	48	11608	0.0208333	0.8447876	1
ko04012	ErbB signaling pathway	Environmental Information Processing	Signal transduction	3	441	100	11608	0.03	0.7378222	1
ko04013	MAPK signaling pathway - fly	Environmental Information Processing	Signal transduction	5	441	129	11608	0.0387597	0.5459442	1
ko04014	Ras signaling pathway	Environmental Information Processing	Signal transduction	11	441	330	11608	0.0333333	0.7146007	1
ko04015	Rap1 signaling pathway	Environmental Information Processing	Signal transduction	10	441	315	11608	0.031746	0.7633742	1
ko04022	cGMP-PKG signaling pathway	Environmental Information Processing	Signal transduction	6	441	229	11608	0.0262009	0.8722601	1
ko04071	Sphingolipid signaling pathway	Environmental Information Processing	Signal transduction	2	441	149	11608	0.0134228	0.9791039	1
ko04072	Phospholipase D signaling pathway	Environmental Information Processing	Signal transduction	6	441	194	11608	0.0309278	0.7515674	1
ko04080	Neuroactive ligand-receptor interaction	Environmental Information Processing	Signaling molecules and interaction	10	441	326	11608	0.0306748	0.7985889	1
ko04110	Cell cycle	Cellular Processes	Cell growth and death	4	441	153	11608	0.0261438	0.838374	1
ko04114	Oocyte meiosis	Cellular Processes	Cell growth and death	1	441	162	11608	0.0061728	0.9981988	1
ko04130	SNARE interactions in vesicular transport	Genetic Information Processing	Folding, sorting and degradation	1	441	38	11608	0.0263158	0.7710369	1
ko04136	Autophagy - other	Cellular Processes	Transport and catabolism	1	441	40	11608	0.025	0.7881592	1
ko04137	Mitophagy - animal	Cellular Processes	Transport and catabolism	3	441	76	11608	0.0394737	0.5557538	1
ko04140	Autophagy - animal	Cellular Processes	Transport and catabolism	5	441	164	11608	0.0304878	0.7518975	1
ko04141	Protein processing in endoplasmic reticulum	Genetic Information Processing	Folding, sorting and degradation	7	441	213	11608	0.0328638	0.7048863	1

Pathway ID	Pathway Name	Level 1	Level 2	Term Candidate Gene Num	Total Candidate Gene Num	Term Gene Num	Total Gene Num	Rich Ratio	P value	Q value
ko04144	Endocytosis	Cellular Processes	Transport and catabolism	12	441	451	11608	0.0266075	0.9279206	1
ko04145	Phagosome	Cellular Processes	Transport and catabolism	5	441	204	11608	0.0245098	0.8919017	1
ko04146	Peroxisome	Cellular Processes	Transport and catabolism	2	441	110	11608	0.0181818	0.9255055	1
ko04150	mTOR signaling pathway	Environmental Information Processing	Signal transduction	2	441	187	11608	0.0106952	0.9942662	1
ko04152	AMPK signaling pathway	Environmental Information Processing	Signal transduction	5	441	153	11608	0.0326797	0.6957749	1
ko04210	Apoptosis	Cellular Processes	Cell growth and death	7	441	189	11608	0.037037	0.581503	1
ko04211	Longevity regulating pathway	Organismal Systems	Aging	2	441	114	11608	0.0175439	0.9343888	1
ko04212	Longevity regulating pathway - worm	Organismal Systems	Aging	3	441	118	11608	0.0254237	0.8313381	1
ko04213	Longevity regulating pathway - multiple	Organismal Systems	Aging	2	441	79	11608	0.0253165	0.8077961	1
ko04214	Apoptosis - fly	Cellular Processes	Cell growth and death	2	441	72	11608	0.0277778	0.7645666	1
ko04217	Necroptosis	Cellular Processes	Cell growth and death	8	441	223	11608	0.0358744	0.6163334	1
ko04218	Cellular senescence	Cellular Processes	Cell growth and death	6	441	212	11608	0.0283019	0.821456	1
ko04260	Cardiac muscle contraction	Organismal Systems	Circulatory system	2	441	86	11608	0.0232558	0.84382	1
ko04261	Adrenergic signaling in cardiomyocytes	Organismal Systems	Circulatory system	4	441	192	11608	0.0208333	0.9374537	1
ko04320	Dorso-ventral axis formation	Organismal Systems	Development	2	441	96	11608	0.0208333	0.8847078	1
ko04330	Notch signaling pathway	Environmental Information Processing	Signal transduction	3	441	78	11608	0.0384615	0.5734942	1
ko04370	VEGF signaling pathway	Environmental Information Processing	Signal transduction	3	441	72	11608	0.0416667	0.5189916	1

Pathway ID	Pathway Name	Level 1	Level 2	Term Candidate Gene Num	Total Candidate Gene Num	Term Gene Num	Total Gene Num	Rich Ratio	P value	Q value
ko04391	Hippo signaling pathway - fly	Environmental Information Processing	Signal transduction	2	441	120	11608	0.0166667	0.9458561	1
ko04520	Adherens junction	Cellular Processes	Cellular community - eukaryotes	5	441	162	11608	0.0308642	0.7423009	1
ko04530	Tight junction	Cellular Processes	Cellular community - eukaryotes	8	441	318	11608	0.0251572	0.9209677	1
ko04540	Gap junction	Cellular Processes	Cellular community - eukaryotes	1	441	107	11608	0.0093458	0.9844499	1
ko04550	Signaling pathways regulating pluripotency	Cellular Processes	Cellular community - eukaryotes	3	441	160	11608	0.01875	0.9458788	1
ko04611	Platelet activation	Organismal Systems	Immune system	12	441	361	11608	0.033241	0.7237169	1
ko04612	Antigen processing and presentation	Organismal Systems	Immune system	2	441	105	11608	0.0190476	0.9128059	1
ko04622	RIG-I-like receptor signaling pathway	Organismal Systems	Immune system	4	441	105	11608	0.0380952	0.5690113	1
ko04624	Toll and Imd signaling pathway	Organismal Systems	Immune system	1	441	66	11608	0.0151515	0.9229735	1
ko04658	Th1 and Th2 cell differentiation	Organismal Systems	Immune system	3	441	123	11608	0.0243902	0.851624	1
ko04664	Fc epsilon RI signaling pathway	Organismal Systems	Immune system	3	441	84	11608	0.0357143	0.6239937	1
ko04666	Fc gamma R-mediated phagocytosis	Organismal Systems	Immune system	5	441	177	11608	0.0282486	0.8078514	1
ko04670	Leukocyte transendothelial	Organismal Systems	Immune system	6	441	171	11608	0.0350877	0.6362747	1
ko04714	Thermogenesis	Organismal Systems	Environmental adaptation	7	441	290	11608	0.0241379	0.9285023	1
ko04720	Long-term potentiation	Organismal Systems	Nervous system	1	441	93	11608	0.0107527	0.9731278	1
ko04721	Synaptic vesicle cycle	Organismal Systems	Nervous system	1	441	73	11608	0.0136986	0.9413624	1
ko04722	Neurotrophin signaling pathway	Organismal Systems	Nervous system	6	441	174	11608	0.0344828	0.6529438	1

Pathway ID	Pathway Name	Level 1	Level 2	Term Candidate Gene Num	Total Candidate Gene Num	Term Gene Num	Total Gene Num	Rich Ratio	P value	Q value
ko04723	Retrograde endocannabinoid	Organismal Systems	Nervous system	6	441	161	11608	0.0372671	0.5774764	1
ko04727	GABAergic synapse	Organismal Systems	Nervous system	4	441	106	11608	0.0377358	0.5765601	1
ko04728	Dopaminergic synapse	Organismal Systems	Nervous system	4	441	175	11608	0.0228571	0.9041106	1
ko04740	Olfactory transduction	Organismal Systems	Sensory system	9	441	1769	11608	0.0050876	1	1
ko04744	Phototransduction	Organismal Systems	Sensory system	2	441	54	11608	0.037037	0.6137623	1
ko04745	Phototransduction - fly	Organismal Systems	Sensory system	1	441	62	11608	0.016129	0.9099878	1
ko04750	Inflammatory mediator regulation of TRP	Organismal Systems	Sensory system	5	441	146	11608	0.0342466	0.6558196	1
ko04810	Regulation of actin cytoskeleton	Cellular Processes	Cell motility	8	441	356	11608	0.0224719	0.9638838	1
ko04910	Insulin signaling pathway	Organismal Systems	Endocrine system	4	441	197	11608	0.0203046	0.9450331	1
ko04911	Insulin secretion	Organismal Systems	Endocrine system	4	441	130	11608	0.0307692	0.7332351	1
ko04912	GnRH signaling pathway	Organismal Systems	Endocrine system	4	441	133	11608	0.0300752	0.749375	1
ko04914	Progesterone-mediated oocyte maturation	Organismal Systems	Endocrine system	1	441	115	11608	0.0086957	0.9886277	1
ko04915	Estrogen signaling pathway	Organismal Systems	Endocrine system	6	441	194	11608	0.0309278	0.7515674	1
ko04916	Melanogenesis	Organismal Systems	Endocrine system	3	441	136	11608	0.0220588	0.8947118	1
ko04917	Prolactin signaling pathway	Organismal Systems	Endocrine system	1	441	90	11608	0.0111111	0.9697885	1
ko04918	Thyroid hormone synthesis	Organismal Systems	Endocrine system	3	441	91	11608	0.032967	0.6775436	1
ko04919	Thyroid hormone signaling pathway	Organismal Systems	Endocrine system	2	441	161	11608	0.0124224	0.9860423	1

Pathway ID	Pathway Name	Level 1	Level 2	Term Candidate Gene Num	Total Candidate Gene Num	Term Gene Num	Total Gene Num	Rich Ratio	P value	Q value
ko04922	Glucagon signaling pathway	Organismal Systems	Endocrine system	4	441	133	11608	0.0300752	0.749375	1
ko04925	Aldosterone synthesis and secretion	Organismal Systems	Endocrine system	4	441	131	11608	0.0305344	0.7386998	1
ko04927	Cortisol synthesis and secretion	Organismal Systems	Endocrine system	1	441	77	11608	0.012987	0.9498294	1
ko04931	Insulin resistance	Human Diseases	Endocrine and metabolic diseases	4	441	130	11608	0.0307692	0.7332351	1
ko04932	Non-alcoholic fatty liver disease (NAFLD)	Human Diseases	Endocrine and metabolic diseases	5	441	180	11608	0.0277778	0.8192348	1
ko04934	Cushing's syndrome	Human Diseases	Endocrine and metabolic diseases	5	441	203	11608	0.0246305	0.8894666	1
ko04940	Type I diabetes mellitus	Human Diseases	Endocrine and metabolic diseases	2	441	76	11608	0.0263158	0.7902093	1
ko04960	Aldosterone-regulated sodium reabsorption	Organismal Systems	Excretory system	1	441	40	11608	0.025	0.7881592	1
ko04961	Endocrine and other factor-regulated calcium	Organismal Systems	Excretory system	2	441	59	11608	0.0338983	0.6618902	1
ko04970	Salivary secretion	Organismal Systems	Digestive system	7	441	200	11608	0.035	0.6411464	1
ko04971	Gastric acid secretion	Organismal Systems	Digestive system	2	441	119	11608	0.0168067	0.9440872	1
ko05010	Alzheimer's disease	Human Diseases	Neurodegenerative diseases	4	441	235	11608	0.0170213	0.9803075	1
ko05014	Amyotrophic lateral sclerosis (ALS)	Human Diseases	Neurodegenerative diseases	2	441	93	11608	0.0215054	0.8736147	1
ko05016	Huntington's disease	Human Diseases	Neurodegenerative diseases	11	441	379	11608	0.0290237	0.8584874	1
ko05034	Alcoholism	Human Diseases	Substance dependence	7	441	228	11608	0.0307018	0.7685664	1
ko05130	Pathogenic Escherichia coli infection	Human Diseases	Infectious diseases: Bacterial	1	441	112	11608	0.0089286	0.9872116	1
ko05131	Shigellosis	Human Diseases	Infectious diseases: Bacterial	4	441	154	11608	0.025974	0.8420316	1

Pathway ID	Pathway Name	Level 1	Level 2	Term Candidate Gene Num	Total Candidate Gene Num	Term Gene Num	Total Gene Num	Rich Ratio	P value	Q value
ko05160	Hepatitis C	Human Diseases	Infectious diseases: Viral	4	441	170	11608	0.0235294	0.8916787	1
ko05164	Influenza A	Human Diseases	Infectious diseases: Viral	9	441	247	11608	0.0364372	0.5989564	1
ko05168	Herpes simplex infection	Human Diseases	Infectious diseases: Viral	15	441	414	11608	0.0362319	0.6130231	1
ko05203	Viral carcinogenesis	Human Diseases	Cancers: Overview	5	441	244	11608	0.0204918	0.9579265	1
ko05214	Glioma	Human Diseases	Cancers: Specific types	2	441	113	11608	0.0176991	0.9322668	1
ko05215	Prostate cancer	Human Diseases	Cancers: Specific types	3	441	117	11608	0.025641	0.8270067	1
ko05216	Thyroid cancer	Human Diseases	Cancers: Specific types	2	441	48	11608	0.0416667	0.5493258	1
ko05218	Melanoma	Human Diseases	Cancers: Specific types	3	441	84	11608	0.0357143	0.6239937	1
ko05221	Acute myeloid leukemia	Human Diseases	Cancers: Specific types	2	441	76	11608	0.0263158	0.7902093	1
ko05223	Non-small cell lung cancer	Human Diseases	Cancers: Specific types	2	441	77	11608	0.025974	0.7962235	1
ko05225	Hepatocellular carcinoma	Human Diseases	Cancers: Specific types	8	441	223	11608	0.0358744	0.6163334	1
ko05231	Choline metabolism in cancer	Human Diseases	Cancers: Overview	4	441	178	11608	0.0224719	0.91095	1
ko05332	Graft-versus-host disease	Human Diseases	Immune diseases	2	441	60	11608	0.0333333	0.6709174	1
