# Supplementary material for: Transcriptome Profile Analysis of Intestinal Upper Villus Epithelial Cells and Crypt Epithelial Cells of Suckling Piglets
Source: Animals (Basel). 2022 Sep 7;12(18):2324. doi: 10.3390/ani12182324 (PMC9494997; doi:10.3390/ani12182324)
Supplement: Supplementary file 1 [file animals-12-02324-s001.zip › Table S4. Primers used for qRT-PCR analysis.pdf]

Table S4. Primers used for qRT-PCR analysis

Gene	Prime	Sequence
HOXA5	Forward	5'- GTT CCG TGA GCG AGC AAT TC -3'
	Reverse	5'- ACG CTG AGA TCC ATG CCA TT -3'
ZFP36	Forward	5'- CAT GGA TCT CAC CGC CAT CT -3'
	Reverse	5'- GCG CTA GAG GGT GAG ACT TG -3'
KLF2	Forward	5'- GAC CAC GAT CCT CCT TGA CG -3'
	Reverse	5'- GCA TCA AGC CTC GAT CCT CT -3'
HSP70-2	Forward	5'- CTT CAA CAT GAA GAG CGC CG -3'
	Reverse	5'- TGA TGG GGT TAC ACA CCT GC -3'
MUC13	Forward	5'- GCT ACA GTG GAG TTG GCT GT -3'
	Reverse	5'- GAC GAA TGC AAT CAC CAG GC-3'
WNT10B	Forward	5'- TGA GTG AGG AGC CCG AAG TT-3'
	Reverse	5'- CAA GGA GGG CTC CGG AAT AG-3'
AQP10	Forward	5'- GAC ACA CGG AAC AAG GGA GT-3'
	Reverse	5'- CCC ACC ACC AAC CAT TAC CA-3'
SLC15A1	Forward	5'- CAG ACT TCG ACC ACA ACG GA-3'
	Reverse	5'- TTA TCC CGC CAG TAC CCA GA-3'
GAPDH	Forward	5'- ATG GTG AAG GTC GGA GTG AA-3'
	Reverse	5'- CGT GGG TGG AAT CAT ACT GG-3'
